# Stem Cell Differentiation as a Non-Markov Stochastic Process

**DOI:** 10.1016/j.cels.2017.08.009

**Published:** 2017-09-27

**Authors:** Patrick S. Stumpf, Rosanna C.G. Smith, Michael Lenz, Andreas Schuppert, Franz-Josef Müller, Ann Babtie, Thalia E. Chan, Michael P.H. Stumpf, Colin P. Please, Sam D. Howison, Fumio Arai, Ben D. MacArthur

**Affiliations:** 1 Centre for Human Development, Stem Cells and Regeneration, Faculty of Medicine, University of Southampton, Southampton SO17 1BJ, UK; 2 Institute for Life Sciences, University of Southampton, Southampton SO17 1BJ, UK; 3 Joint Research Center for Computational Biomedicine, RWTH Aachen University, 52056 Aachen, Germany; 4 Aachen Institute for Advanced Study in Computational Engineering Science, RWTH Aachen University, 52062 Aachen, Germany; 5 Maastricht Centre for Systems Biology, Maastricht University, 6229 ER Maastricht, the Netherlands; 6 Zentrum für Integrative Psychiatrie, Universitätsklinikum Schleswig-Holstein Campus Kiel, Niemannsweg 147, 24105 Kiel, Germany; 7 Max Planck Institute for Molecular Genetics, Ihnestraße 63-73, 14195 Berlin, Germany; 8 Centre for Integrative Systems Biology and Bioinformatics, Department of Life Sciences, Imperial College, London SW7 2AZ, UK; 9 Mathematical Institute, University of Oxford, Oxford OX2 6GG, UK; 10 Department of Stem Cell Biology and Medicine, Graduate School of Medical Sciences, Kyushu University, Fukuoka 812-8582, Japan; 11 Mathematical Sciences, University of Southampton, Southampton SO17 1BJ, UK

**Keywords:** statistical mechanics, stem cells, lineage commitment, single-cell biology, stochastic process, non-Markov process

## Abstract

Pluripotent stem cells can self-renew in culture and differentiate along all somatic lineages *in vivo*. While much is known about the molecular basis of pluripotency, the mechanisms of differentiation remain unclear. Here, we profile individual mouse embryonic stem cells as they progress along the neuronal lineage. We observe that cells pass from the pluripotent state to the neuronal state via an intermediate epiblast-like state. However, analysis of the rate at which cells enter and exit these observed cell states using a hidden Markov model indicates the presence of a chain of unobserved molecular states that each cell transits through stochastically in sequence. This chain of hidden states allows individual cells to record their position on the differentiation trajectory, thereby encoding a simple form of cellular memory. We suggest a statistical mechanics interpretation of these results that distinguishes between functionally distinct cellular “macrostates” and functionally similar molecular “microstates” and propose a model of stem cell differentiation as a non-Markov stochastic process.

## Highlights

• We profile individual stem cells as they differentiate along the neural lineage
• Regulatory network changes and increased cell variability accompany differentiation
• Analysis of dynamics with a hidden Markov model reveals unobserved molecular states
• We propose a model of stem cell differentiation as a non-Markov stochastic process

## Graphical Abstract

**Figure:**
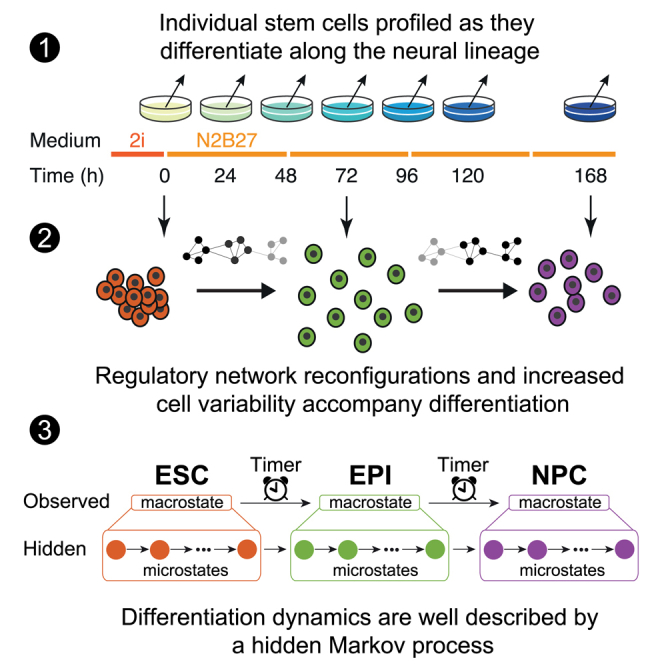

## Teaser

We profile individual mouse embryonic stem cells as they progress along the neuronal lineage. Analysis of observed cell dynamics using a hidden Markov model reveals the presence of a chain of unobserved molecular states that each cell transits through stochastically in sequence. We suggest a statistical mechanics interpretation of these results and propose a model of stem cell differentiation as a non-Markov stochastic process.

## Introduction

Two distinct pluripotent states are found in the pre-gastrulation mouse embryo: a naive pluripotent state that emerges from the inner cell mass of the blastocyst between E3.5 and E4.5 and a primed pluripotent state that emerges after implantation of the blastocyst into the uterus wall at E5.5 (Nichols and Smith, 2009). During this naive-to-primed pluripotency transition, cells undergo dramatic changes to their signaling requirements, transcriptional regulatory control mechanisms, and global epigenetic status (Nichols and Smith, 2009). These molecular changes are accompanied by morphological changes of the pluripotent tissue *in vivo* (Tam and Loebel, 2007).

Following this transition, cells become increasingly susceptible to the spatially coded differentiation cues that determine the foundation of the principal germ layers in the body. A variety of molecular mechanisms regulate this susceptibility in order to prevent premature lineage commitment and enable the correct formation of the egg cylinder, including the regionalization of the extra-embryonic endoderm and hence the foundation for the formation of differential signaling gradients across the embryo during gastrulation (Tam and Loebel, 2007).

At this stage, the timely release of pluripotency maintenance mechanisms is just as important as the gain of lineage-specific characteristics (Betschinger et al., 2013, Nichols and Smith, 2009, Turner et al., 2014), and appropriate differentiation is regulated by the balance of these two processes. However, despite recent interest in this problem (Moris et al., 2016, Semrau et al., 2016, Hormoz et al., 2016), the dynamics of exit from the pluripotent state at the individual cell level are only partially understood.

In particular, while it is known that stochastic fluctuations in key transcription factors have an important role in the early stages of differentiation (Chambers et al., 2007, Toyooka et al., 2008, Hayashi et al., 2008, Abranches et al., 2014), it is not yet clear if cellular responses to these fluctuations are also stochastic or if this inherent molecular stochasticity is buffered and differentiation progresses in a deterministic way through a continuum of intermediary cell states (MacArthur et al., 2012, Moris et al., 2016, Semrau et al., 2016, Hormoz et al., 2016).

Previous reports have sought to approach these issues by using mathematical and computational models to dissect the structure and function of the gene regulatory networks that underpin specific cell identities and differentiation events (Müller et al., 2008, MacArthur et al., 2012, MacArthur et al., 2009, Dunn et al., 2014) or by considering differentiation in more abstract terms using notions from dynamical systems theory, for example as a noise-induced or driven transition between attractor states (Ridden et al., 2015, Chang et al., 2008, Mojtahedi et al., 2016, Richard et al., 2016, Furusawa and Kaneko, 2012).

Both of these approaches have advantages and disadvantages: the first focuses on details and therefore aims to provide understanding of the molecular mechanisms that regulate specific cell-fate transitions, yet relies either on possession of a good *a priori* understanding of key molecular drivers or a robust way to infer them from data, and is not well equipped to separate lineage-specific details from more general mechanisms that may be active in other contexts. By contrast the second focuses on principles, and therefore aims to provide a general way to understand cell-fate transitions in the absence of detailed molecular regulatory information yet is not well equipped to dissect the specifics of any particular fate transition.

Here, we sought to combine these two approaches by profiling a well-defined transition in detail, and then using a range of different mathematical modeling and analysis methods to examine the resulting data. Using this integrative approach, we explore how pluripotency regulatory networks are reconfigured during the early stages of embryonic stem cell (ESC) differentiation along the neural lineage and propose a general view of stem cell lineage commitment that uses notions from statistical mechanics to distinguish between unobserved internal molecular “states” and observable cell “types.”

## Results

### Differentiation *In Vitro* Recapitulates Developmental Dynamics *In Vivo*

Starting from the pluripotent ground state in leukemia inhibitory factor (LIF) + 2i conditions, the closest *in vitro* equivalent to the naive pluripotent state of the pre-implantation epiblast (Ying et al., 2008), we directed differentiation of mouse ESCs in mono-layer culture toward the neuroectoderm using a well-established protocol (Ying et al., 2003, Bain et al., 1996). This transition was chosen since it has previously been shown to induce robust and reliable differentiation (Ying et al., 2003, Abranches et al., 2009) and therefore serves as a good model system to examine the kinetics of the exit from pluripotency and the gain of acquired lineage characteristics.

**Figure 1:**
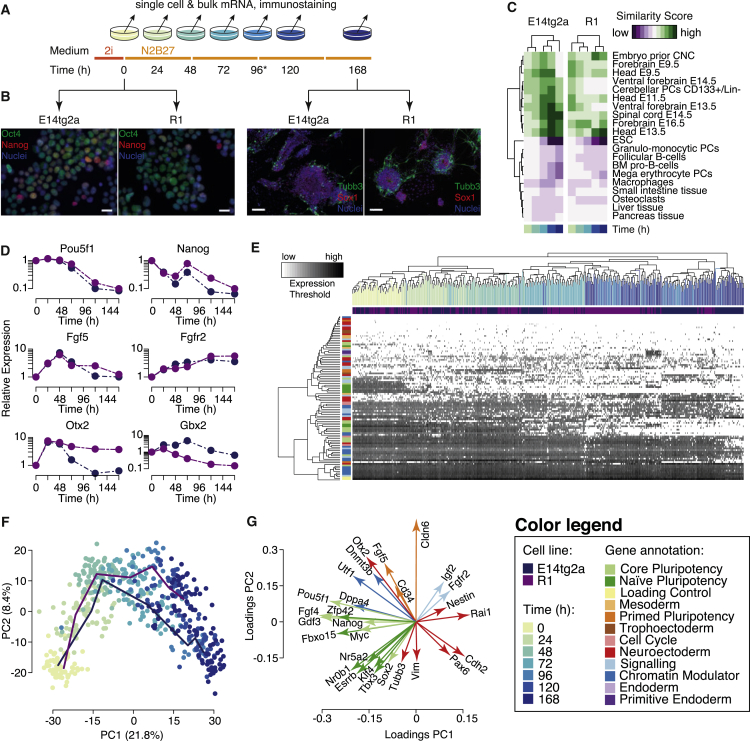
Differentiation *In Vitro* Recapitulates Development *In Vivo* (A) Schematic of the experimental design. (B) Immunostaining for pluripotency markers Oct4 and Nanog from cells at the start of the experiment (left panels, scale bars 50 μm) and neuronal markers Tubb3b and Sox1 at the end of the experiment (right panels, scale bars 200 μm). (C) Comparison of global gene expression profiles with a training library shows loss of pluripotency characteristics and progressive gain of neuronal characteristics. Comparisons with the 20 most similar/dissimilar lineages are shown. The full comparison is shown in Figure S2. (D) Loss of pluripotency markers and gain of neuronal lineage markers assessed by RT-PCR. (E) Single-cell data show a gradual drift from the ESC state to the NPC state. (F) Projection of the data onto the first two principal components reveals the presence of a transient intermediate state during differentiation. Color indicates sampling time. Solid lines show mean trajectories for each cell line. (G) Gene loadings for the first two principal components indicates that the intermediate state is a primed epiblast-like state. Throughout this figure, data for the R1 cell line are given in blue, and data for the E14 cell line are given in purple.

To determine the global molecular dynamics of differentiation, mRNA expression changes were assessed via microarray of bulk cell material, and morphological and protein expression changes were examined by immunostaining (Figure 1A). To extract general rather than cell-line-specific processes, we conducted two biological replicates, starting with ESCs derived from mice with different genetic backgrounds (R1 and E14tg2a [E14] strains).

We observed that in both cases, cells of the starting population abundantly expressed proteins related to the pluripotent state (Figures 1B, S1A, and S1B), while at the final time point of the differentiation trajectory (168 hr), cells were marked primarily by neuronal stem cell marker *Sox1* and early neuronal marker *Tubb3* (Figures 1B, S1C, and S1D), indicating a predominantly neuroprogenitor cell (NPC) phenotype.

To better understand the dynamics of the transition from the ESC state to the NPC state, we constructed a supervised machine-learning classifier that compares the observed gene expression patterns with those from a training library of 161 cell-type-specific gene expression profiles curated from the literature (for complete list, see Table S1) and produces a similarity score for each lineage based upon our previously published methodology (Lenz et al., 2013).

This analysis revealed a gradual loss over time of gene expression characteristics associated with pluripotency and early development, and a sequential emergence of gene expression patterns related to the neural tube and brain development, in accordance with the appropriate mouse developmental stages (Figures 1C and S2 and Table S2). In particular, we observed that gene expression patterns became increasingly similar to those seen during specific stages of the head and ventral forebrain development (E9.5–E16.5), while similarity to tissues of mesodermal and endodermal origin was either consistently low or progressively reduced over time.

Complementary analysis of global gene expression changes identified 1,726 differentially expressed genes throughout the time course with substantial overlap between the two cell lines (Figures S1F–S1H). Among those 877 consistently upregulated genes, annotation terms for brain tissue and neuron differentiation were significantly over-represented (*p* = 8.1 × 10^−3^ and *p* = 2.9 × 10^−8^ false discovery rate [FDR] corrected, respectively), while annotations for ESC and stem cell maintenance were enriched among the 849 downregulated genes (*p* = 1.7 × 10^−3^ and *p* = 8.9 × 10^−3^ FDR corrected, respectively) (Table S3).

These results indicate the induction of appropriate, and broadly similar, differentiation programs in both cell lines. However, subtle differences in gene expression changes between cell lines were also apparent, indicating the initiation of slightly different developmental programs. For instance, expression of *Otx2*, a transcription factor expressed in both primed pluripotent cells (Acampora et al., 2013) and in the developing anterior brain (Simeone et al., 1992), occurred only transiently during the first 48 hr of differentiation in E14 cells, while expression was sustained in R1 cells (Figure 1D). Concomitant with this, expression of *Gbx2*, an antagonist *Otx2* during the formation of the mid/hindbrain junction (Millett et al., 1999, Broccoli et al., 1999), was subsequently induced in E14 but not in R1 cells (Figure 1D), suggesting a slight specification bias intrinsic to each cell line (see also Figure S1I).

These minor differences notwithstanding, taken together these analyses indicate that differentiation *in vitro* reliably recapitulates developmental dynamics *in vivo*.

### Differentiation Progresses through an Intermediary Metastable State

**Figure 2:**
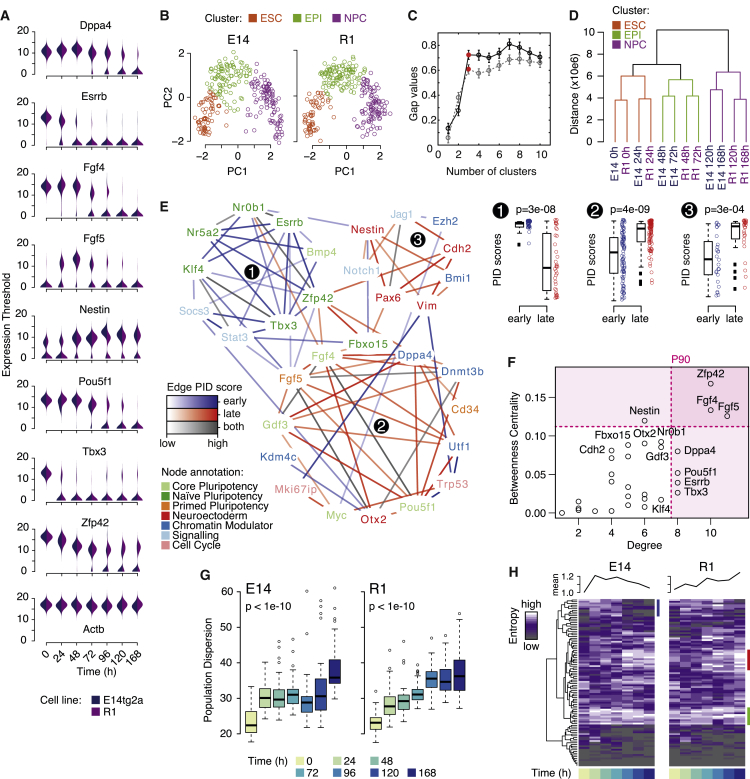
Differentiation Is Accompanied by Regulatory Network Re-configurations and an Increase in Cell-Cell Variability (A) Bean plots of expression changes of key genes from single-cell RT-PCR data. (B) Single cell expression data naturally cluster into three distinct groups. Data are projected onto the first two principal components, determined independently for each cell line. Color indicates classification according to *k*-means clustering with three clusters. (C) Assessment of cluster quality using the GAP statistic (Tibshirani et al., 2001). The most natural partition of the data is associated with the “elbow” in this plot, here at three clusters highlighted in red. Bars show SEs. Data for the E14 cell line is in black; data for the R1 cell line is in gray. (D) Microarray expression data also naturally clusters into three groups. (E) Regulatory network inferred from single-cell data has three distinct modules that are active at different times during differentiation. Boxplots to the right show the distributions of PID scores, which measure edge importance (see STAR Methods), for all edges in each cluster at early and late times; all the data points are shown beside the boxes. Significant changes in PID scores indicate differential expression of the module over time. p values were obtained using a Wilcoxon rank-sum test. (F) Genes with high degree are likely important for consolidating cellular identities in each state. Genes with high betweenness centrality are likely important in the transition between states. Dotted lines show the 90th percentile. (G) Cell-cell variability, as assessed by multivariate dispersion (see STAR Methods), increases over the time course. p values were obtained using a Wilcoxon rank-sum test. (H) Shannon entropy, as a measure of gene expression variation, increases monotonically in the R1 cell line and transiently in the E14 cell line. Heatmaps show entropy changes for all genes measured; line plots show mean entropy over all genes measured at each time point. Side bars show genes that increase in variability in the middle of the time course (green); at the end of the time course (red); and those that lose variability at the end of the E14 time course (blue). In all boxplots, boxes show first and third quartiles about the median, whiskers extend to 1.5 times the interquartile range from the box. Data points beyond whiskers are shown as outliers above or below boxes.

To investigate the dynamics of differentiation further, we sought to monitor differentiation dynamics at the single-cell level. To do so, gene expression changes for 96 pre-selected genes of interest (including regulators of pluripotency and neuronal differentiation, as well as epigenetic and cell-cycle regulators, see Table S4) were recorded periodically over the course of the time series within individual cells using a high-throughput RT-PCR array (Figures 1A, 1E, and 2A).

Hierarchical clustering of the data largely captured the natural ordering by sampling time, indicating a gradual progression of cellular identities away from the ESC state toward the NPC state (Figure 1E).

Dimensionality reduction using principal component (PC) analysis suggested that cells do not move directly from the ESC state to the NPC state but rather pass through a transitory intermediate state characterized by particular combinatorial patterns of gene expression (Figure 1F). Analysis of the contribution of the gene loadings to each of the first two PCs revealed that the dynamics may be decomposed into two distinct molecular processes (Figure 1G): PC1 associates with the transition from the ground state of pluripotency toward the neuronal lineages (regulators of the ground state ESC identity such as *Pou5f1*, *Nanog*, *Esrrb*, *Zfp42*, *Klf4*, *Tbx3*, *Nr0b1*, and *Myc* are negatively associated with this component; while genes associated with the NPC identity such as *Nestin*, *Rai1*, *Pax6*, and *Cdh2* are positively associated); while PC2 associates with the process of epiblast maturation (regulators of the primed epiblast that forms the egg cylinder, such as *Otx2*, *Fgf5*, *Cd34*, and *Cldn6* as well as generic epigenetic regulators such as *Utf1* and *Dnmt3b* are positively associated with this component; while characteristic neuronal genes such as *Vim* and *Tubb3* are negatively associated) (see Figure 1G).

These analyses affirm similar dynamics seen in previous studies (Abranches et al., 2009, Boroviak et al., 2014, Kalkan and Smith, 2014) and indicate that differentiation progresses through three phenotypically distinct cell states: from the ground state of pluripotency to a primed epiblast-like state before the commitment to neural lineage is specifically made.

To further determine if this partition into three states is a strong feature of the data, we also conducted *k*-means clustering for 2–10 clusters and analyzed cluster qualities using the GAP statistic, a simple metric that compares the within-cluster variability present for a given clustering to that expected from appropriate randomization (Hastie et al., 2001), in order to identify natural clustering patterns in the data. This analysis revealed the presence of three robust clusters in the data (characterized by naive pluripotency, epiblast, and neural progenitor markers, respectively) and thereby confirmed that the biologically intuitive partition of differentiation into three distinct phases is a natural feature of data (Figures 2B–2D and S3A).

Taken together, this analysis suggests that ESC differentiation along the neuronal lineage progresses via two transitions through three biologically distinct cell states.

### Cell-State Changes Are Accompanied by Regulatory Network Reconfigurations

Having identified three robust cell states, we wanted to better understand the transcriptional changes that occur as cells move from one state to another and to identify functional relationships between genes that mediate these transitions. We reasoned that if two genes are co-regulated, or if one gene regulates the other, then we would observe coordinated changes in the expression levels of these genes over time. We therefore sought to infer a putative regulatory network from the data in order to better understand any patterns in these coordinated changes.

Here, by the term “regulatory network,” we refer to the set of (co-)regulatory relationships between genes that are active under a given experimental condition or at a specific developmental stage, rather than the complete set of all possible physical gene-gene interactions that are hardwired in the genome. These relationships will, of course, vary over time as cells progress through development, resulting in re-configuration of the inferred network structure.

To infer regulatory network reconfigurations, we assumed that gene interactions that are actively involved in driving developmental progression would result in observable changes in cell transcriptional states and induce statistical dependencies in the expression patterns of the interacting genes. To identify these coordinated changes, we made use of information-theoretic measures that enable the identification of non-linear statistical relationships between variables (here, genes), and are therefore substantially more powerful than traditional correlation-based network inference approaches (McMahon et al., 2014). In particular, we used the partial information decomposition (PID), a recently derived method to examine the statistical relationships between three or more variables that provides a more detailed description of statistical relationships than standard information-theoretic measures such as pairwise mutual information (Williams and Beer, 2010).

Our PID-based algorithm assigns a score to each potential gene-gene interaction, indicating the strength of statistical association, which we interpret to be evidence of a putative functional relationship, and selects only those interactions that pass a stringent selection criterion. A full discussion of our method may be found in the companion paper to this article, also published in this edition of *Cell Systems* (Chan et al., 2017). Summary details are provided in the STAR Methods.

This analysis revealed a network enriched with connections between known regulators of pluripotency and neuronal differentiation (Figure 2E). To dissect how regulatory interactions change over time, we applied this method to different subsets of the data: to infer interactions important for the early stages of differentiation, we used data from cells identified as being in the ESC and epiblast-like (EPI) states; to identify interactions important for the later stages of differentiation, we used data from cells identified as being in the EPI or NPC states (individual cells were identified as being in the ESC, EPI, or NPC state via *k*-means clustering, as described above). We selected these subsets comprising pairwise combinations of cell states to ensure that each subset includes cells at a variety of stages of the developmental transition in question (either ESC to EPI, or EPI to NPC), providing the heterogeneity necessary to detect statistical dependencies between observed gene expression states. This analysis revealed strong clustering of edges according to their temporal importance (as colored in Figures 2E and S3B).

To investigate this clustering further, we then identified regulatory modules within the network using an unsupervised community detection algorithm that identifies modules across different scales without assuming a fixed number of modules in advance (Delvenne et al., 2010).

This analysis revealed the presence of seven regulatory modules (Figure S3B), three of which displayed significant changes in activity over time (Figure 2E). Genes in module 1 are primarily associated with the ground state of pluripotency (see Table S4 for gene annotations) and reduce substantially in expression during the early stages of differentiation. Genes in module 2 are primarily associated with the primed epiblast-like state and are generally transiently upregulated toward the middle of the time series and downregulated from approximately 72 hr onward. Genes in module 3 are primarily associated with neuroectoderm differentiation, and generally increase in expression throughout the time course.

While most genes within each of these three modules primarily display strong intra-module connectivity (that is, they connect strongly to other members of the same module but weakly to members of different modules), some genes such as *Zfp42*, *Fgf5*, *Fgf4*, and *Nestin* also showed high inter-module connectivity (as assessed by betweenness centrality, a simple measure of node importance; see Newman (2010) and STAR Methods), suggesting a potential role for these genes in coordinating the transitions between states (Figure 2F). In contrast, those genes that form the hubs of their respective modules, such as *Esrrb*, *Tbx3*, *Dppa4*, and *Pou5f1* (Figure 2F) may be involved in the maintenance or consolidation of one particular cell state.

Collectively, these results reaffirm that the early stages of differentiation progress through two distinct pluripotent states and indicate that coordinated changes in regulatory network structure accompany these cell-state changes.

### Gene Expression Variability Increases during Differentiation

Once we had identified these three states, we sought to better understand the dynamics of cellular transitions between states. We reasoned that if cells pass from one state to another in a coordinated deterministic way at a constant rate, then the initial cell-cell variability present in the population would propagate with time and therefore remain approximately constant through the time series. Alternatively, if cells progress in an uncoordinated, stochastic way from one state to another, then cell-cell variability would increase over time.

To investigate this, we estimated the total dispersion within the population at each time point from the single-cell expression data. Dispersion is a multivariate measure of cell-cell variability that takes into account the variability of each gene as well as the patterns of covariance between genes (see STAR Methods). This analysis revealed a significant increase in cell-cell variability over time (Figure 2G).

To investigate this increase further, we also estimated the Shannon entropy of expression for each gene at each time point, as a simple measure of expression variability (MacArthur and Lemischka, 2013, Richard et al., 2016). We found that while some genes remain relatively homogeneously expressed throughout the experiment (see bottom cluster in Figure 2H), others showed substantial changes in variability. Typically, these changes occurred either immediately upon the exit from pluripotency and persisted through the middle of the time course (highlighted with green side bar in Figure 2H), or arose in the latter stages of commitment (highlighted with red side bar in Figure 2H). Patterns of variation were generally consistent between the two cell lines (compare the two heatmaps in Figure 2H, which use the same gene ordering), indicating that the observed changes in gene expression variability are intrinsic characteristics of the differentiation process.

To investigate how global patterns of variability changed over time, we also calculated the mean entropy of gene expression at each time point in both cell lines. We observed a general increase in mean entropy as differentiation progressed in the R1 cell line, and a transient increase at the exit from pluripotency in the E14 cell line (Figure 2H).

While the reason for this disparity is not fully clear, it appears to reflect slight differences in the kinetics of the entry to the neuronal state. For example, there is a panel of genes—which includes some important regulators of pluripotency including *Pou5f1* (also known as *Oct4*), *Nanog*, and *Lif*, as well as *Otx2*, a regulator of both the primed pluripotent state and anterior brain development—that become more homogeneously expressed at the end of the time course in the E14 cell line, yet are relatively heterogeneously expressed in the R1 line (see blue highlight bar in Figure 2H).

This difference in variability relates to differences in the loss of expression of these genes in the two cell lines. For instance, although expression of *Pou5f1* is quickly lost between 72 and 96 hr in most cells from both cell lines (see Figure 2A), a small residual population of cells retained *Pou5f1* expression in the R1 line to 168 hr, while expression was entirely lost in the E14 line beyond 96 hr. These differences suggest that the E14 cell line consolidates the neural progenitor identity slightly earlier than the R1 line, and this earlier consolidation is revealed as a loss of cell-cell variability toward the end of the E14 experiment.

Taken together, these analyses indicate that cell-cell variability increases upon the exit from the pluripotent state. It is likely that the early increase in cell-cell variability is due to a stochastic response to the release of the stringent constraints that 2i culture conditions impose on the cells. Since similar increases in cell-cell variability have been observed during differentiation in other mammalian systems (Richard et al., 2016, Mojtahedi et al., 2016, Semrau et al., 2016), it may also reflect more generic mechanisms such as the “flickering” that is often found in stochastic systems passing through a critical point (Scheffer et al., 2009).

These results indicate that while all cells are exposed to the same differentiation cues, cellular differentiation in response to these cues progresses in an uncoordinated and apparently stochastic way.

### A Stochastic Model of Stem Cell Differentiation

In summary, our statistical analysis confirmed the widely accepted model that differentiation progresses through three functional cell states: from the initial ESC state, to a primed EPI state, and then on to the final NPC state (Abranches et al., 2009, Boroviak et al., 2014, Kalkan and Smith, 2014).

However, the increase in cell-cell variability we observed also indicated that cells do not synchronize their transitions through these states. Rather it appeared that individual cells progress in an uncoordinated, stochastic manner. We reasoned that this inherent stochasticity might be important, yet the mechanisms by which it is regulated were not clear.

To investigate further, we sought to construct a series of mathematical models to explore the process of differentiation further (see Box 1 for details). To do so, we first assigned each cell in the time course to either the ESC, EPI, or NPC state based upon our cluster analysis (see above, Figure 2B and STAR Methods). Since each cell also comes with a time label (the time at which it was sampled), we were able to use these two labels to monitor and model how the proportion of cells in the ESC, EPI, and NPC states changed over time as differentiation progressed.

Box 1 **Mathematical Models**

**Figure 3:**
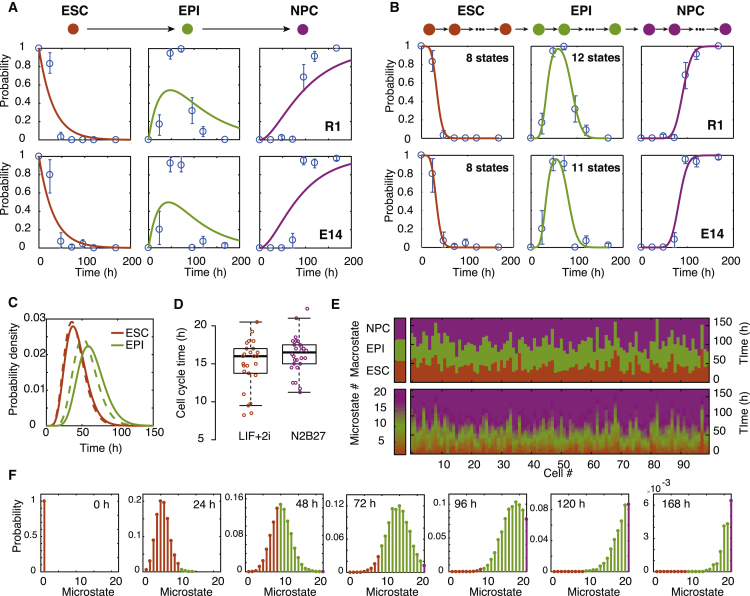
Data Fitting to a Hidden Markov Model Reveals the Presence of Cellular Microstates (A) Fit of data to (Equation 1), (Equation 2), (Equation 3). Data are in blue; mean and 95% confidence intervals about the mean from bootstrapped *k*-means clustering are shown. This memoryless stochastic process does not describe the data well. (B) Fit of data to Equation 10. Data are in blue; mean and 95% confidence intervals about the mean from bootstrapped *k*-means clustering are shown. Data are well described by this stochastic process with memory. (C) Wait-time distribution in the ESC and EPI states. Full lines show E14 data, dotted lines show the R1 data. (D) Cell-cycle times in LIF + 2i and N2B27 media are significantly longer than the inferred microstate residence times. Boxes show 1st and 3rd quartiles about the median, whiskers extend to 1.5 times the interquartile range from the box. Data points beyond whiskers are shown as outliers above or below boxes. (E) Illustrative simulation of 100 cells according to our hidden Markov model, given in Equations 7, 8, and 10. Parameters are taken from the R1 model fit. (F) The resulting evolving probability density function over the microstates colored by macrostate. Throughout this figure, orange represents the ESC state; green represents the EPI state; and purple represents the NPC state.

Let *p*_*A*_(*t*), *p*_*B*_(*t*), and *p*_*C*_(*t*) be the probabilities that a randomly selected cell is in the ESC, EPI, or NPC state, respectively, at experimental time *t*. Assuming that all cells within a given state behave in the same way and transitions between states occur independently at constant average rates, these dynamics are described by the following set of equations:

(Equation 1) $$\frac{dp_{A}}{dt}=-q_{1}p_{A}\text{,}$$

(Equation 2) $$\frac{dp_{B}}{dt}=q_{1}p_{A}-q_{2}p_{B}\text{,}$$

(Equation 3) $$\frac{dp_{C}}{dt}=q_{2}p_{B}\text{,}$$

where *q*_1_ and *q*_2_ are transition probabilities per unit time, and we assume that *p*_*A*_(0) = 1 and *p*_*B*_(0) = *p*_*C*_(0) = 0 (i.e., all cells start in the ESC state). This model, which assumes that cells within each observable state are homogeneous with respect to their differentiation potential, does not describe the data well (see Figure 3A).

This suggests that either: (1) cells do not transit independently at a constant average rate from one state to the next, but rather transition rates are affected by paracrine feedback mechanisms within the developing colony; or (2) individual cells within each observable state are not interchangeable, but rather are distinguished from one another with respect to some intrinsic hidden variables.

**Figure 4:**
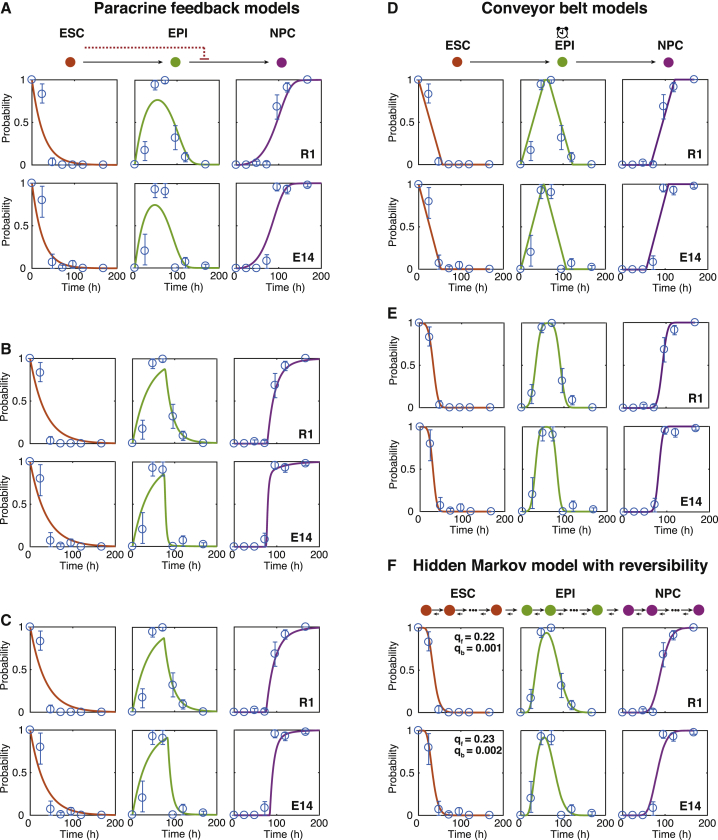
Fits of Mathematical Models to the Data Full details of all models are given in Box 1 and the STAR Methods. In all panels, data are in blue; mean and 95% confidence intervals about the mean from bootstrapped *k*-means clustering are shown. (A) Paracrine feedback model without cooperativity (Hill coefficient, *h* = 1). (B) Paracrine feedback model with unconstrained Hill coefficient. (C) Paracrine feedback model with ultrasensitivity (*h*→∞). (D) Conveyor-belt model with uniform initial conditions. (E) Conveyor-belt model with Gaussian initial conditions. (F) Hidden Markov model with reversible dynamics. Inferred forward transition probabilities (*q*_*f*_) per unit time are approximately 100–200 times larger than reverse transition probabilities (*q*_*b*_), indicating that differentiation is a strongly directional process.

A natural variation that accounts for the first option is to allow residual undifferentiated ESCs in the population to inhibit the further differentiation of cells from the EPI to NPC state. Details of this model are given in the STAR Methods. Although this is a plausible mechanism, we found that it does not describe the data well (see Figures 4A–4C), suggesting that paracrine effects are not primarily responsible for the deviation from first-order kinetics that we observe.

To account for the second option, we modified the first-order model to allow each observable “macrostate” to conceal a directed chain of “microstates” (see Box 2 for detailed definitions of microstates and macrostates). Let *p*_*n*_ be the probability that a cell is at microstate *n* at time *t*. For simplicity, we assume that the cells transition independently from one microstate to the next on average at the same rate and transitions are irreversible. In this case, the dynamics of the hidden Markov process are given by

(Equation 4) $$\frac{dp_{0}}{dt}=-qp_{0}\text{,}$$

(Equation 5) $$\frac{dp_{n}}{dt}=q\left(p_{n-1}-p_{n}\right)\;\;\text{for }n=1\ldots N-1\text{,}$$

(Equation 6) $$\frac{dp_{N}}{dt}=qp_{N-1}\text{.}$$

where *q* is the transition probability per unit time, with *p*_*n*_(0) = *δ*_*n*0_, where *δ* is the Kronecker delta function (i.e., all cells start in the first microstate), and we have assumed that the chain contains *N*+1 microstates in total.

This model is simply a homogeneous Poisson process, and may be solved exactly to give

(Equation 7) $$p_{n}\left(t\right)=\frac{{\left(qt\right)}^{n}}{n!}{\text{e}}^{-qt}=f\left(n\text{;}qt\right)\;\;\text{for }0\leq n<N\text{,}$$

(Equation 8) $$p_{N}\left(t\right)=1-\overset{N-1}{\sum_{n=0}}p_{n}\left(t\right)\text{,}$$

where *f*(*n*;*qt*) is the Poisson probability density function.

Assuming that microstates 0, 1, 2,…, *n*_*A*_ identify with the ESC state, microstates *n*_*A*_ + 1, *n*_*A*_ + 2,…, *n*_*B*_ identify with the EPI state, and microstates *n*_*B*_ + 1, *n*_*B*_ + 2,…, *N* identify with the NPC state, the observed probabilities,

(Equation 9) $$p_{A}\left(t\right)=\sum_{n=0}^{n_{A}}p_{n}\left(t\right)\text{,}\;\;p_{B}\left(t\right)=\sum_{n=n_{A}+1}^{n_{B}}p_{n}\left(t\right)\text{,}\;\;p_{C}\left(t\right)=\sum_{n=n_{B}+1}^{N}p_{n}\left(t\right)\text{,}$$

may also easily be found as

(Equation 10) $$p_{A}\left(t\right)=F\left(n_{A}\text{,}qt\right)\text{,}\;\;p_{B}\left(t\right)=F\left(n_{A}\text{,}qt\right)-F\left(n_{B}\text{,}qt\right)\text{,}\;\;p_{C}\left(t\right)=1-F\left(n_{B}\text{,}qt\right)\text{,}$$

where *F*(*n*;*qt*) is the Poisson cumulative distribution function. The dynamics of this model are illustrated in Figures 3E and 3F.

The assumption of reversibility in the microscopic dynamics may be relaxed at the expense of introducing an extra model parameter. Doing so does not substantially improve model fit (see Figure 4F) and results in estimates of forward transition rates that are approximately 100–200 times larger than reverse transition rates, indicating that differentiation is a strongly directional process. Details of a reversible version of this model are given by (Equation 14), (Equation 15), (Equation 16) in the STAR Methods.

A central feature of our hidden Markov model is that it allows cell-cell variability to develop due to the inherently stochastic nature of the differentiation process. However, it is also plausible that at least some of the variation seen during differentiation is due to deterministic propagation of initial cell-cell variability. Details of a closely related continuum model that accounts for this “conveyor-belt”-like process are also given in the STAR Methods. This model is also able to explain the data well, although at the expense of a larger number of free parameters (see Figures 4D and 4E and Discussion).

**Figure B1:**
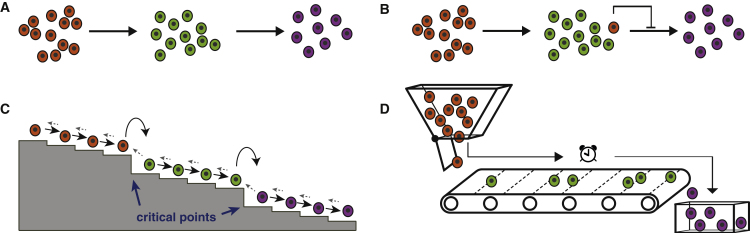
Schematic of Mathematical Models We consider four classes of model: (A) differentiation obeys first-order kinetics. This model is given by (Equation 1), (Equation 2), (Equation 3) in the text. (B) Differentiation from the EPI state to the NPC state is inhibited by residual ESCs in the colony. This model is given by (Equation 11), (Equation 12), (Equation 13) in the STAR Methods. (C) Differentiation is described by a hidden Markov process. This model is given by (Equation 4), (Equation 5), (Equation 6) and 10 in the text. A minor variation to allow reversible dynamics is given by (Equation 14), (Equation 15), (Equation 16) and 17 in the STAR Methods. (D) Differentiation is described by a continuous “conveyor-belt” process in which initial variability propagates forward at constant speed. This model is given by Equation 18 in the STAR Methods. In all panels, orange denotes cells in the ESC state; green denotes cells in the EPI state; purple denotes cells in the NPC state.

A schematic illustrating all of the models we considered is given in Figure B1.

In our first, most basic, model we assumed that cells are initially held in the naive pluripotent state when cultured in 2i conditions, yet once these extrinsic constraints are released, cells progress stochastically from one state to the next at constant average rates (see schematic in Figure 3A and details in Box 1). We found that this first model does not describe the data well (Figure 3A), since it allows cells to transition quickly through the ESC, EPI, and NPC states, yet we observed that the first pioneer neurons emerge *in vitro* only after 72–96 hr (Figure S1C), corresponding to the same phenomenon in mouse corticogenesis from E8.5 onward (Stainier and Gilbert, 1990). Thus, while the majority of cells accumulate in the EPI state around 72 hr in experiments, the model cannot account for this accumulation.

This suggested that individual cells within each state are not interchangeable with respect to their differentiation potential, but rather are distinguished from one another with respect to some hidden (that is, unmeasured) variables. To better understand the observed dynamics, we therefore constructed a range of alternative mathematical models that took into account both cell-intrinsic and cell-extrinsic hidden mechanisms (see Box 1 and STAR Methods for details).

We found that cell-extrinsic mechanisms did not explain well the deviation from first-order kinetics that we observed (see Figures 4A–4C). However, a simple hidden Markov model that uses ideas from statistical mechanics (Pathria, 1996) to distinguish between unobserved molecular states internal to the cell and observable cell identities did perform well (Figure 3B).

In this revised model, we allowed the observed ESC, EPI, and NPC “macrostates” to conceal a directed chain of hidden “microstates,” which the cells transit through stochastically in sequence at a constant average rate (see Box 2, Box 3 for detailed definitions of microstates and macrostates and Box 1 for further model details). While these microstates are not directly observable, their presence can be inferred by considering the rates at which cells enter and exit the observed macrostates.

Box 2 **Statistical Mechanics**

Differentiation is the process by which cells with specialist function are produced from less specialized founder cells. Since differentiation is the transition from an unspecialized to a specialized cell type, understanding differentiation requires a robust notion of what a cell “type” is. Notably, despite tremendous recent progress in dissecting the molecular basis of cell-fate decisions, this is still a subject of considerable debate (Cell Systems, 2017).

In practice, cell types are often characterized by distinct functions or morphologies, or by distinct patterns of gene or protein expression. However, there is no *a priori* reason why these two definitions should be directly related: many internal molecular states (i.e., patterns of gene/protein expression, etc.) may map to the same cell function and different functions may be performed by cells with similar internal molecular states. It is likely that there is a complex, interdependent relationship between the inherently stochastic molecular dynamics that occur within individual cells and the emergence of well-defined cell fates. Indeed, how robust and reproducible cell identities emerge from the fog of molecular noise is one of the great, and still largely mysterious, wonders of cell biology.

This interdependence between the molecular and the cellular is reminiscent of similar problems encountered in statistical mechanics, and recent years have seen interest in using ideas from statistical mechanics to better understand cell fates (Garcia-Ojalvo and Martinez-Arias, 2012, Trott et al., 2012, MacArthur and Lemischka, 2013, Moris et al., 2016).

**Figure B2:**
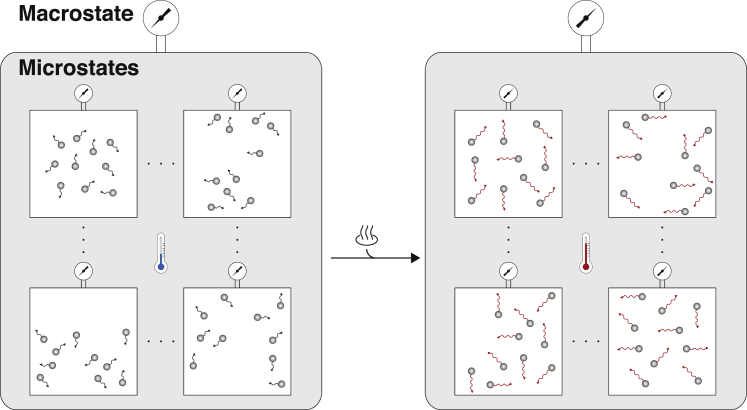
Microstates and Macrostates Statistical mechanics makes a clear distinction between the bulk properties of matter (known as macrostates) and internal molecular configurations (known as microstates). For example, the pressure of a gas in confinement is a macrostate that arises from collisions of the gas molecules with the walls of the container. Importantly, full knowledge of the position and momentum of each molecule in the gas is not needed to measure its pressure: for a fixed number of molecules and a fixed volume only the average kinetic energy per molecule is needed and, subject to reasonable assumptions on the dynamics, any molecular configuration with the same average will give rise to the same pressure (left panel). As the temperature of the gas is raised, the average kinetic energy per molecule is increased and the pressure increases accordingly (right panel). Typically macroscopic system properties change continuously with control parameters; however, at certain critical points (see Box 3) system properties may change abruptly.

Statistical mechanics is the branch of physics that seeks to understand how macroscopic properties of matter, such as pressure, density, etc., arise from the microscopic dynamics of the atoms and molecules of which matter is composed (see Box 3). It has been shown that a clear distinction between macrostates (bulk properties) and microstates (internal molecular configurations) is both possible and advantageous. A fundamental principle of statistical mechanics is that each macrostate corresponds to a large number of interchangeable microstates. The fundamental triumph of statistical mechanics was to show, in a rigorous way, how many of the observable macroscopic properties of matter emerge naturally and reproducibly from the stochastic evolution of the ensemble of microstates (see Pathria, 1996 and Figure B2).

Our model of differentiation aims to begin to apply some of these ideas to a specific biological context. By analogy with statistical mechanics, we assume that each microstate is a distinct molecular configuration internal to the cell, broadly defined to include patterns of gene/protein expression and expression and activity of epigenetic regulators, etc. By contrast, each macrostate is a distinct functional cell “type,” in this case the ESC, EPI, and NPC identities. This formalism is similar to that proposed in Trott et al. (2012) and Moris et al. (2016).

As in statistical mechanics, we allow many different microstates to map to the same macrostate (i.e., we endow cells in different molecular states with the ability to perform the same function) and allow stochastic transitions between microstates to take place. In principle, microstates may be arranged in a complex geometry and conversion back and forth between microstates within each macrostate may occur. Indeed, a central principle of standard statistical mechanics is that microscopic dynamics are reversible at equilibrium, a concept known as detailed balance. However, here, since cells are being driven away from the ESC state and toward the NPC state, the system is fundamentally out of equilibrium, and so is not expected to obey detailed balance.

To account for the non-equilibrium nature of the dynamics, we take the simplest possible arrangement of microstates: they are ordered in a directed chain, and contiguous blocks are associated with successive macrostates. By doing so, we are assuming that during the process of differentiation, the rate of forward transitions greatly exceeds the reverse rate to the extent that reverse transitions do not significantly affect differentiation dynamics. Full details of the model are given in Box 1 and a variation of the model to allow reverse transitions is also considered in Figure 4F.

Box 3 **Glossary**

**Statistical mechanics.** The branch of physics that uses probability theory to study how large-scale properties of matter emerge via averaging from the inherently stochastic dynamics of the elements of which matter is composed.
**Microstate.** A complete description of the position and momenta (or other relevant property) of every particle in a system.
**Macrostate.** A macroscopic property of a system, e.g., the pressure of a gas in confinement.
**Detailed balance.** A fundamental principle of equilibrium statistical mechanics, which states that at equilibrium each forward process is equilibrated by its reverse.
**Critical point.** The value of a control parameter at which some observable system property changes qualitatively.

To estimate the number of hidden microstates within the ESC and EPI states, we therefore fit this model to the data, including a regularization term that penalizes excessive numbers of microstates (see STAR Methods for details). Model fitting indicated the presence of 8 hidden microstates within the observed ESC state for both cell lines and 11 (12) microstates within the observed EPI state for R1 cells (E14 cells, respectively). The expected transition time between microstates was 5.3 (4.8) hr for R1 cells (E14 cells, respectively), giving a mean residence times of 42.6 (40.8) hr in the ESC state, and 63.9 (56.1) hr in the EPI state for R1 cells (E14 cells, respectively) (Figure 3C).

It is of note that these inferred transition times between microstates are significantly shorter than the cell-cycle time, which is approximately 15 hr for these cells in both 2i and N2B27 media (Figure 3D), while the inferred transition times between macrostates are significantly longer than the cell-cycle time. This suggests that the dynamics are not primarily driven by cell division events but rather by some other, as yet unidentified, molecular processes. In principle, since our modeling framework deliberately does not make the nature of cellular microstates explicit, transitions between microstates may be associated with any putative molecular processes.

Candidates for driving mechanisms include the range of cell-intrinsic processes that are known to be important for lineage commitment, such as alterations in DNA methylation (Meissner et al., 2008, Habibi et al., 2013, Singer et al., 2014, Lee et al., 2014) and other global chromatin state changes (Mikkelsen et al., 2007, Ziller et al., 2015); varying promoter dynamics (Miyanari and Torres-Padilla, 2012, Deng et al., 2014); and transcriptional (Marks et al., 2012) and post-transcriptional regulation (Salomonis et al., 2010) of gene expression.

Furthermore, although we found that they do not appear to drive the observed dynamics, cell-extrinsic processes related to the local microenvironment (van den Brink et al., 2014, Bedzhov and Zernicka-Goetz, 2014) and various forms of cell-cell communication (Habib et al., 2013, Dunn et al., 2014) are also likely to be important. Indeed, it is probable that macroscopic transitions are associated with the collective action of multiple cell-intrinsic and -extrinsic molecular processes, for example, via engagement of fate-determining feedback loops, to direct changes in cell identities.

The notable consistency in the fitted model parameter values between the two cell lines suggests that both lines are undergoing a common dynamical process, despite their slight molecular differences. This consistency indicates that, although complex and inherently stochastic, the underlying microscopic dynamics are regulated and reproducible, and therefore amenable to further investigation. Such analysis is beyond the scope of this paper, but could, for example, utilize live-cell tracking to follow individual cells as they progress through differentiation.

Taken together, this analysis suggests that stem cell differentiation along the neuronal lineage is a strongly canalized yet inherently stochastic process.

## Discussion

Recent years have seen remarkable advances in high-throughput single-cell profiling technologies (Shapiro et al., 2013). To better understand the data that these new and emerging methods produce, there is now a need for modeling and analysis methods that sift functional cell-cell variability from measurement noise and identify distinct cellular identities from highly heterogeneous data.

These issues are particularly apparent when considering time course data, and a number of computational tools have accordingly recently been developed to explore single-cell fate trajectories and cell-cell variability within heterogeneous populations (Stegle et al., 2015). These computational models are typically based on the assumption that cells progress continuously through measurable cell states and so implicitly assume that underlying molecular stochasticity is buffered to the extent that a continuum approximation is appropriate. However, it has been observed that combinatorial fluctuations in key lineage-specifying factors are important for stem cell fate specification (Chambers et al., 2007, Toyooka et al., 2008, Hayashi et al., 2008, MacArthur and Lemischka, 2013, Abranches et al., 2014), and it has accordingly been argued that cell-fate commitment is a discrete stochastic process (Moris et al., 2016).

Here, we have outlined an alternative modeling framework that infers the presence of discrete hidden cell states from limited expression data and have used this framework to dissect the dynamics of neuronal differentiation of mouse ESCs *in vitro*.

In accordance with previous observations, we find that differentiation progresses through two functionally distinct pluripotent cell states: a naive pluripotent state representative of the transient ESC state *in vivo* and a primed pluripotent state, representative of the post-implantation epiblast *in vivo* (Abranches et al., 2009, Boroviak et al., 2014). However, we also found that cell-cell variability increased over time, suggesting that differentiation is an inherently stochastic process.

To better understand this stochasticity, we considered a simple model in which these observed states conceal a multitude of functionally similar hidden molecular states. By analogy with statistical mechanics (MacArthur and Lemischka, 2013, Garcia-Ojalvo and Martinez-Arias, 2012, Moris et al., 2016), we refer to the observable functional cell states as cellular macrostates and the variety of molecular configurations associated with each functional macrostate as molecular microstates (see Box 2).

In our framework, the microscopic dynamics are given by a homogeneous Poisson process in which the number of hidden states is allowed to vary. Since the probability that a cell will transition to the next microstate per unit of time is independent of how long it has spent in its current microstate, this underlying stochastic process is Markovian (or memoryless). However, transitions between macrostates are not Markovian; the probability that a cell will move to the next macrostate depends on how long it has already spent in the current macrostate.

**Figure 5:**
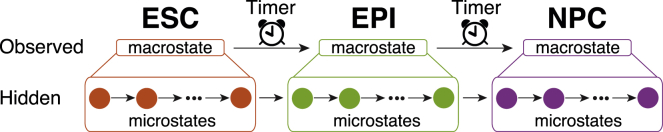
Schematic of Model Framework Cells transition at a constant rate through a chain of hidden microstates, which are not directly observed but rather group together into observable macrostates and act to time transitions between macrostates. While the underlying dynamics are Markovian, the observable dynamics are non-Markovian, and may therefore be thought of as a stochastic process with memory.

Thus, the macroscopic dynamics, which describe transitions between functional cell types, are formally a stochastic process with “memory” (see Figure 5 for a schematic, and STAR Methods for details of the equations describing the macroscopic dynamics). During differentiation, this memory is important since it allows individual cells to keep a record of their progress and provides a simple mechanism by which cells can consolidate a particular functional identity before progressing onto the next. In our view, the interplay between microstates and macrostates and the resulting non-Markovian nature of the macroscopic dynamics are central to the regulation of differentiation.

For example, the number of microstates along the differentiation chain has an important role in regulating its output. In the case of differentiation of mouse ESCs along the neuronal lineage, we estimate that there are 20–21 states in the chain (Figure 3B). Thus, while each transition from one microstate to the next is inherently stochastic, a large number of these transitions must occur in order for the cell to differentiate fully.

In stochastic analysis, it is well known that the output of such a chain of stochastic events becomes less variable as the length of the chain increases, a result that is known as the law of large numbers (Gardiner, 1985). In our model, this means that the length of time it takes for an individual cell to complete the differentiation trajectory becomes less variable as the number of microstates on the trajectory increases. The large number of microstates we estimate in the chain between the ESC and the NPC states therefore serves to regularize an inherently stochastic process and ensure that differentiation occurs in a reliable and reproducible way.

Although the model that we propose describes the data well, a number of unresolved questions remain.

Firstly, while our current framework is deliberately agnostic regarding the molecular processes that drive differentiation, we observed remarkable consistency between cell lines suggesting that, although inherently stochastic, differentiation is a precisely regulated dynamical process at the single-cell level. In principle, the details of single-cell differentiation dynamics should be amenable to further analysis. As methods for live-cell tracking and analysis develop, we are hopeful that specifics will added to our sketch. Ultimately, a consolidated model of differentiation at the individual cell level will require detailed understanding of the stochastic dynamics of underlying molecular regulatory networks and will necessarily draw upon techniques from a range of different areas, including stochastic analysis and dynamical systems theory (Furusawa and Kaneko, 2012).

Secondly, while our model seeks to examine how cell-cell variability develops within an initially homogeneous population, it is likely that at least some of the variation seen during the differentiation process is due to deterministic propagation of initial cell-cell variability. Indeed, we found that such a conveyor-belt-like process is also able to explain our data well, albeit at the expense of a larger number of free parameters (see Box 1 and Figures 4D and 4E). However, since in this model the variation present in the population remains constant (by construction), it cannot account for the transient increase in cell-cell variability during differentiation that we observed (see Figures 2G and 2H).

By contrast, a transient increase in cell-cell variability is inherent to the hidden Markov model that we propose. This may be seen directly from Equation 10 or informally by noting that in this model the cell population starts and ends in a homogeneous state (initially all cells are in the first microstate and the final NPC-associated microstate is absorbing), yet each cell traverses the chain of microstates stochastically, thereby generating transient heterogeneity in the population.

In practice, it is likely that the dynamic cell-cell variation that we observe during differentiation results from a range of different interacting mechanisms, including uncertainty in initial conditions. Dissecting these interactions should provide fruitful work for the coming years.

In summary, our analysis indicates stem cell differentiation is a highly regulated stochastic process that is amenable to analysis using the tools of statistical mechanics. We anticipate that some of the most exciting future advances in stem cell science will combine new experimental techniques with further theoretical developments in the physics of living matter.

## STAR★Methods

### Key Resources Table

**Table:** 

REAGENT or RESOURCE	SOURCE	IDENTIFIER
**Antibodies**		

anti-Oct-3/4 Antibody (C-10)	Santa Cruz	sc-5279; RRID: AB_628051
anti-Nanog Antibody	abcam	ab80892; RRID: AB_2150114
anti-Sox2 Antibody	Santa Cruz	sc-17320; RRID: AB_2286684
anti-Sox1 Antibody (H-85)	abcam	ab87775; RRID: AB_2616563
anti-Sox17 Antibody	R&D	AF1924; RRID: AB_355060
anti-betaIII Tubulin Antibody (2G10)	abcam	ab78078; RRID: AB_2256751
Goat anti-Mouse IgG (H+L) Antibody	life technologies	A-11017; RRID: AB_143160
Donkey anti-Rabbit IgG (H+L) Antibody	Pierce Antibodies	SA5-10040; RRID: AB_2556620

**Biological Samples**		

Mouse embryonic fibroblasts	Prepared in house	N/A

**Chemicals, Peptides, and Recombinant Proteins**		

SUPERase⋅ In™ RNase Inhibitor	life technologies	AM2694
Leukemia inhibitory factor (LIF)	produced in house	N/A
MEK inhibitor (PD0325901)	Tocris bioscience	4192
GSK-3 inhibitor (CHIR99021)	Reagents Direct	27-H76
Paraformaldehyde	Sigma Aldrich	P6148
Triton-X-100	Sigma Aldrich	X100
Fetal bovine serum	life technologies	10270106
4’,6- diamidino-2-phenylindole	Sigma Aldrich	D9542
DMEM	life technologies	31053-028
KnockOut serum replacement	life technologies	35050-038
non-essential amino acids	life technologies	11140-050
GlutaMax	life technologies	10828-010
DMEM/F12 medium	life technologies	21041025
Neurobasal medium	life technologies	12348017
B27 supplement	life technologies	17504044
N2 supplement	life technologies	17502048
Trypsin/EDTA	PAA	L11-003
Penicillin/Streptomycin	PAA	P11-10
Gelatine	Sigma Aldrich	G1890
2-mercaptoethanol	Sigma Aldrich	M6250

**Critical Commercial Assays**		

CellsDirect One-Step qRT-PCR Kit	life technologies	11753
AllPrep DNA/RNA Mini Kit	Quiagen	80204
Fluidigm 96x96 Dynamic Array kit	Fluidigm	BMK-M10-96.96
MouseWG-6 v2.0 Expression BeadChip Microarrays	Illumina	BD-201-0602

**Deposited Data**		

Single cell qPCR data	This paper	http://dx.doi.org/10.17632/g2md5gbhz7.1
Microarray data	This paper	ArrayExpress: E-MTAB-5861
Microarray data used for similarity score.	see Table S1	see Table S1

**Experimental Models: Cell Lines**		

ES-E14tg2a	Gift from Neil Smyth, University of Southampton	N/A
ES-R1	Gift from Neil Smyth, University of Southampton	N/A

**Oligonucleotides**		

A full list of oligonucleotides used in this study is provided as Table S4.		see Table S4

**Software and Algorithms**		

NIS elements v4.3 software	Nikon UK	
Matlab (version 8.5 or later)	MathWorks	https://www.mathworks.com
R (version 3.1.2 or later)		https://www.r-project.org
*lumi*	Du et al. (2008) 24(13):1547-1548. Bioinformatics.	http://bioconductor.org/packages/release/bioc/html/lumi.html
Affymetrix Power Tools	Affymetrix	http://www.affymetrix.com/estore/partners_programs/programs/developer/tools/powertools.affx
Bayesian Blocks Algorithm	Scargle et al. (2013)	
Regulatory network inference	Chan et al. (2017)	

### Contact for Reagent and Resource Sharing

Further information and requests for resources and reagents should be directed to and will be fulfilled by the Lead Contact, Ben D. MacArthur (bdm@soton.ac.uk).

### Experimental Model and Subject Details

#### Routine Cell Culture

Pluripotent mouse embryonic stem cell lines R1 (Nagy et al., 1993) and E14tg2a (Kuehn et al., 1987, Doetschman et al., 1987) were obtained from Neil Smyth, Southampton University, Southampton, UK. Cells were cultivated in Dulbecco’s Modified Eagle Medium (DMEM; life technologies, Paisley, UK, #31053-028) with 1% Penicillin/Streptomycin (PAA, Yeovil, UK, #P11-10) that was further supplemented with 15% KnockOut serum replacement, 1x MEM non-essential amino acids, 1x GlutaMax (all from life technologies, Paisley, UK, #10828-010, #11140-050 and #35050-038), 50 *μ*M 2-mercaptoethanol (Sigma Aldrich, Gillingham, UK, #M6250). Leukaemia inhibitory factor (LIF), produced in house, was added at a saturating dilution of 1:1000. Cells were seeded on 0.1% gelatine (Sigma-Aldrich, Gillingham, UK, Cat. No. G1890) coated tissue culture plates pre-seeded with *γ*-irradiated MEF for routine culture. Throughout four subsequent passages prior to the start of the experiment, cells were cultivated in 0.1% gelatine coated tissue culture plates without additional MEF, and medium was additionally supplemented with a combination of 1 *μ*M PD0325901 (Tocris bioscience, #4192) and 10 *μ*M CHIR99021 (Reagents Direct, #27-H76). Cells were maintained at 37°C and 5% CO_2_ and routinely passaged every other day using Trypsin/EDTA (PAA, Yeovil, UK, #L11-003). Medium was replaced on a daily basis.

#### Neuronal Differentiation

Neuronal differentiation medium (N2B27) was prepared according as previously described (Ying et al., 2003) and contained a mixture of Neurobasal and DMEM/F12 media, supplemented with B27 and N2 supplements (Thermo Fisher, Cat.No. 12348017, 21041025, 17504044 and 17502048).

### Method Details

#### Isolation of mRNA

Total mRNA was isolated from cell lysates according to manufacturer’s instructions using the AllPrep DNA/RNA Mini Kit (Quiagen, Crawley, UK, Cat.No. 80204).

#### Global Gene Expression Microarrays

For global gene expression, total mRNA isolated from ensemble cells was processed and hybridized to MouseWG-6 v2.0 Expression BeadChip mircoarrays by CGS genomics, Cambridge, UK. Pre-processing of raw expression data was performed in R (version 3.1.2 or later) using the *lumi* package (Du et al., 2008) and the robust spline normalization method. Differentially expressed genes (DEG) were identified based on total expression changes relative to 0h across all time points, denoted as cumulative relative expression (CRE). A gene was considered a DEG when it’s CRE surpassed a threshold of 3 times the interquartile range above (below) the 75 percentile (25 percentile) based on the entirety of CREs.

#### Single Cell Gene Expression Arrays

Individual cells were sorted using a BD FACS Aria II flow cytometer into 96-well round bottom multi-well plates (both Becton-Dickinson, Oxford, UK). Single cells were de-posited directly into 5 *μ*l of reaction mix containing reagents for cell lysis, reverse transcription, as well as the polymerase and reaction buffers for RT-PCR. The reaction mix consisted of 0.1 *μ*l Superscript III RT/Platinum Taq Mix, 0.05 *μ*l Ambion’s SUPERase12-In, 1.85 *μ*l DEPC-treated water (all part of CellsDirect One-Step qRT-PCR Kit, life technologies, Paisley, UK, Cat. No. 11753) and 0.0125 *μ*l of 96 different TaqMan assays (probe IDs included in Table S4) for multiplex pre-amplification. The reverse transcription and pre-amplification was performed on a Veriti thermal cycler (life technologies, Paisley, UK) with the following temperature cycles: 15 min, 50°C; 2 min, 95°C followed by 22 cycles of 15 s at 95°C alternating with 1 min at 60°C. Thus, pre-amplified cDNA was diluted with 20 *μ*l of DEPC-treated water and stored at -80°C until further processing. Readout was performed using Fluidigm 96x96 Dynamic Array in combination with the Biomark HD system (both Fluidigm, San Francisco, USA) according to manufacturers instructions. Cycling threshold (CT) values ≥ 28 were considered absent. Raw CT values were normalized using the median CT values of loading controls (*Actb* and *Gapdh*) for each array. Normalised CT values were then transformed linearly to expression threshold (ET) values ranging from 0 (absent) to 28 (maximum expression). Cells with low readings for loading controls (CT > 15), and low or high overall expression (ET < *Q*_1_ − 2*I*, and ET > *Q*_3_ + 2*I*, where *Q*_*i*_ is the *i*th quartile and *I* is the interquartile range) were excluded.

#### Immunofluorescence Staining

Cells were fixed for 20 min at room temperature (RT) using 4% Paraformaldehyde (Sigma-Aldrich, Gillingham, UK, #P6148) in PBS-/- (PAA, Yeovil, UK, #H15-002) and washed three times with PBS-/-. Intracellular epitopes were made accessible by permeabilisation of the cell and nuclear membranes using a 0.2% Triton-X-100 (Sigma-Aldrich, Gillingham, UK, #X100) solution in PBS-/-for 10min at RT. Unspecific binding sites were blocked for 45min at RT with 0.1% Triton-X-100 and 10% fetal bovine serum (life technologies, Paisley, UK, #10270106) in PBS-/-, washed three more times before re-suspension in blocking buffer and either primary antibody or matching isotype controls and incubation over night at 4°C under slow, continuous agitation. Cells were subsequently washed three times using blocking solution and re-suspended in blocking solution and secondary antibodies for incubation under continuous agitation for 1 h at RT. Samples were washed three times in blocking solution and nuclei were stained at RT for 10 min using 4’,6- diamidino-2-phenylindole (DAPI; Sigma-Aldrich, Gillingham, UK, #D9542) at a concentration of 10 *μ*g/ml. Following a final wash in PBS-/-, cells were imaged using an AxioVert 200 microscope (Carl Zeiss, Cambridge, UK).

#### Cell Cycle Time Analysis

Bright field images of cells grown at 37°C and 5% CO2 in either in 2i+LIF culture medium or N2B27 medium were taken in 15 min intervals using an Eclipse-Ti microscope and NIS elements v4.3 software (both Nikon UK, Kingston Upon Thames, UK). Cell cycle time was measured manually by tracking the number of frames between two subsequent cell division events.

#### Experimental Design

Experimental data were acquired for two biological replicates (embryonic stem cell lines E14tg2a and R1).

Strategy for randomization and/or stratification: not applicable.

Blinding at any stage of the study: not applicable.

Sample-size estimation and statistical method of computation: not applicable.

Inclusion and exclusion criteria of any data or subjects: individual samples in the single-cell expression data were filtered as described in the section on *Single cell gene expression arrays* above.

### Quantification and Statistical Analysis

#### Machine Learning of Cell Identities

To determine how the expression patterns of the cells in our time-course related to known tissues and cell types, we collated a database of 161 tissue/cell type specific expression patterns (Table S1). Raw data sets were downloaded from the Gene Expression Omnibus (GEO, http://www.ncbi.nlm.nih.gov/geo/) database and pre-processed as a single set using the robust multi-array average (RMA) normalization method in the Affymetrix Power Tools software (http://www.affymetrix.com/estore/partners_programs/programs/developer/tools/powertools.affx). The annotation of samples into tissue/cell types was performed manually based on the experimental descriptions in the GEO database. Our experimental data collected at 24h, 48h, 72h, 120h, and 168h from both cell lines (E14 and R1) were compared to the undifferentiated (0h) samples of the respective cell line and expression differences were projected onto the training set as described in (Lenz et al., 2013). Briefly, for each comparison of time points, two gene sets consisting of the top 5% of upregulated genes and top 5% of downregulated genes were defined, and their expression values in each of the 161 tissue/cell type specific expression patterns were compared using a Wilcoxon rank sum test. This resulted in 161 tissue/cell type specific scores per time point for each cell line (signed log10 p values of Wilcoxon test), which summarize the similarity of the observed gene expression pattern with each of the 161 tissue/cell line samples we collated. Overall these evolving scores describe the differentiation dynamics in a genome-wide expression space with physiologically relevant signatures.

#### Clustering and Dimensionality Reduction

All clustering and dimensionality reduction was performed in R (version 3.1.2 or later) and Matlab (version 8.5 or later) using standard routines. We found that a more robust clustering was obtained from the single cell data by taking a binary representation of the data (i.e. retaining only information on whether each gene is expressed or not) and performing PCA, retaining the first 2 components, prior to classification using *k*-means clustering. PCA is a well-established method for data de-noising (Hastie et al., 2001) and discretization of gene expression data has been shown to improve the robustness of subsequent analysis algorithms (Tuna and Niranjan, 2010). Here, these de-noising steps make the subsequent analyses more stable but do not affect any of the conclusions of the paper. The changes in the proportions of cells in each macrostate over time were determined by calculating the fraction of cells in each cluster at each time point. Confidence intervals on proportions were obtained by Bootstrap resampling.

#### Regulatory Network Inference

Normalized single-cell data for each gene were discretized independently using the Bayesian Blocks algorithm, a method designed to find an optimal binning for a set of values without enforcing uniform bin width (Scargle et al., 2013). Data from both cell types (R1 and E14) were combined for this discretization step. There were 22 genes with no detected expression in greater than 80% of cells; data from these genes were removed, leaving 74 genes for all subsequent analyses.

To infer statistical dependencies between genes from the time-series data we developed an information-theoretic network inference algorithm. Many network inference algorithms exist that use the mutual information between pairs of variables as a measure of statistical dependency (McMahon et al., 2014). Here, we adapted these methods to calculate a score between pairs of genes that takes into account the context of the wider network, by considering the multivariate relationships of each pair of genes with every other gene in the network. This method highlights the strongest relationships for each gene, rather than simply the strongest relationships within the whole network. We find that this methods performs better than or comparably to existing information theoretic based inference methods. Full details of this algorithm, along with bench-marking against alternative methods, may be found in an accompanying paper (Chan et al., 2017).

Briefly, we make use of the partial information decomposition (PID) (Timme et al., 2014) to calculate a set of multivariate information measures that encode the statistical relationships between triplets of genes, by decomposing mutual information into synergistic, redundant, and unique contributions. Specifically, if we consider the information provided by a set of genes, e.g. *A* = {*X*,*Y*}, about another target variable, e.g. *Z*, the mutual information *I*(*X*,*Y*;*Z*) between the set *A* and *Z* is equal to the sum of four partial information terms,

*I*(*X*,*Y*;*Z*) = Synergy(*Z*;*X*,*Y*) + Unique(*Z*;*X*) + Unique(*Z*;*Y*) + Redundancy(*Z*;*X*,*Y*)

The mutual information between a single gene (*X*, say) in *A* and the target comprises a unique and redundant contribution,

*I*(*X*;*Z*) = Unique_*Y*_(*Z*;*X*) + Redundancy(*Z*;*X*,*Y*).

For any pair of genes, *X* and *Z*, this mutual information, *I*(*X*;*Z*), is constant regardless of the choice of the third variable, *Y*, but the unique contribution to this information varies with *Y*. Higher ratios of unique information to mutual information indicate a stronger dependency between *X* and *Z* (Chan et al., 2017). Our inference algorithm defines a measure *u*_*X*,*Z*_, based on these ratios, which we call the proportional unique contribution,

$$u_{X,Z}=\sum_{Y\in S\setminus \left\{X,Z\right\}}\frac{{\text{Unique}}_{Y}\left(X;Z\right)}{I\left(X;Z\right)}\;+\sum_{Y\in S\setminus \left\{X,Z\right\}}\frac{{\text{Unique}}_{Y}\left(Z;X\right)}{I\left(X;Z\right)}\text{,}$$

and uses this metric to assess the strength of the relationship between the pair of genes *X* and *Z*, in the context of all the other genes in the network, Y∈S∖{X,Z} (where *S* is the complete set of genes). These proportional unique contributions are then used to calculate a confidence score *c*, which we call the PID score, between each pair of genes,

*c* = *F*_*X*_(*u*_*X*,*Y*_) + *F*_*Y*_(*u*_*X*,*Y*_),

where *F*_*X*_(⋅) is a cumulative distribution function estimated using all the proportional unique contribution scores involving gene *X*. The PID scores are then used as edge weights in the (un-directed) inferred network. Edges were retained in the network if they were in the top 5 % of PID scores.

To identify molecular regulatory mechanisms active at different stages of differentiation we inferred networks from the early part of the time-course (using expression patterns from cells identified as being in the ESC or EPI states) and from the late part of the time-course (using expression patterns from cells identified as being in the EPI or NPC state).

#### Identification of Modules in Regulatory Networks

In order to identify modules within the inferred networks that show coordinated changes in gene expression, we used a community detection method based on the evolution of a Markov process on a network, as described previously (Delvenne et al., 2010). We scanned for stable partitions at 200 Markov times from 10^−2^ to 10^2^, and selected as stable partitions those in which the number of modules remained constant for at least 10 time points, and that corresponded to a minimum in the variation of information.

#### Network Analysis

Let *A*_*ij*_ = *A*_*ji*_ be the adjacency matrix for the network *G*. The degree of node *i* is given by $\sum_{j}A_{ij}$. The betweenness centrality of node *i* is given by $\sum_{j\neq i\neq k}\sigma_{jk}\left(i\right)/\sigma_{jk}$, where $\sigma_{jk}$ is total number of shortest paths between nodes *j* and *k* and $\sigma_{jk}\left(i\right)$ is the total number of shortest paths from nodes *j* to *k* that pass through node *i* (Newman, 2010).

#### Estimation of Dispersion and Entropy

To the ith cell in the population we associate a gene expression vector $G_{i}=\left(g_{i1},g_{i2},\ldots ,g_{i96}\right)\in R^{96}$, which records its expression status with respect to the 96 genes we measured. Assuming that there are *n* cells in the population, the mediancentre is that point $M=\left(m_{1},m_{2},\ldots ,m_{96}\right)\in R^{96}$ such that $D=\sum_{i=1}^{n}d\left(G_{i},M\right)$ is minimum, where $d\left(x,y\right)=\sum_{j=1}^{96}\left|x_{j}-y_{j}\right|$ is the *L*_1_-distance. The mediancentre is a multivariate generalization of the univariate median (Gower, 1974). The dispersion of each cell is its distance to mediancentre *d*(*G*_*i*_,*M*), and the dispersion of the population is the minimized value of *D*. The dispersion is a simple statistic that can be used in hypothesis testing to compare the multivariate variability in different populations.

To estimate gene expression entropy, normalized single-cell data for each gene were discretized independently using the Bayesian Blocks algorithm, a method designed to find an optimal binning for a set of values without enforcing uniform bin width (Scargle et al., 2013). The Shannon entropy, $H=-\sum_{i}P_{i}{\text{log}}_{2}P_{i}$, where *P*_*i*_ is the probability of observing gene expression in bin *i*, was then calculated directly.

#### Model Fitting

For all fits model parameters were estimated by minimizing the residual sum of squares between the data and the model. For continuous problems fitting was performed using the Levenberg-Marquardt algorithm. Since the hidden Markov model has both integer and real parameters optimization for this problem was performed using a pattern search algorithm, implemented in MATLAB (The MathWorks, Natick, MA, 2016) as part of the Global Optimization Toolbox. Models with a large number of microstates generally fitted the data better than those with a small number of microstates, since they effectively introduce more parameters into the model. To avoid over-fitting we therefore penalizing models with large numbers of microstates. Thus, we solved

$$\underset{n_{A},n_{B},q}{\text{min}}{\left|\left|y-f\right|\right|}_{2}+\lambda \left(n_{A}+n_{B}\right)\text{,}$$

where *y* is the data and *f* is the model. The regularization parameter *λ* was selected using the L-curve method (Lawson and Hanson, 1995).

#### Paracrine Feedback Model

To account for paracrine feedback we allow residual ESCs in the population to inhibit further differentiation. A simple model to account for this mechanism is:

(Equation 11) $$\frac{dp_{A}}{dt}=-q_{1}p_{A}\text{,}$$

(Equation 12) $$\frac{dp_{B}}{dt}=q_{1}p_{A}-\frac{q_{2}K^{h}}{K^{h}+p_{A}^{h}}p_{B}\text{,}$$

(Equation 13) $$\frac{dp_{C}}{dt}=\frac{q_{2}K^{h}}{K^{h}+p_{A}^{h}}p_{B}\text{.}$$

This model has four free parameters. Assuming non-cooperative dynamics (*h* = 1) or ultrasensitive dynamics (*h*→∞, in which case the $q_{2}K^{h}p_{B}/\left(K^{h}+p_{A}^{h}\right)\to q_{2}p_{B}H\left(K-p_{A}\right)$, where *H* is the Heaviside step function) reduces the number of parameters to three. None of these variations fit the data well (see Figures 4A–4C) suggesting that paracrine feedback mechanisms are not primarily responsible for the deviation from first order kinetics that we see.

#### Hidden Markov Model with Reversibility

The dynamics along a chain of microstates in which both forward and reverse transitions are allowed are given by

(Equation 14) $$\frac{dp_{0}}{dt}=-q_{f}p_{0}+q_{b}p_{1}\text{,}$$

(Equation 15) $$\frac{dp_{n}}{dt}=q_{f}\left(p_{n-1}-p_{n}\right)+q_{b}\left(p_{n+1}-p_{n}\right)\;\;\text{for }n=1\ldots N-1\text{,}$$

(Equation 16) $$\frac{dp_{N}}{dt}=q_{f}p_{N-1}\text{.}$$

where *q*_*f*_ is the forward transition probability per unit time, and *q*_*b*_ is the backward transition probability per unit time, with *p*_*n*_(0) = *δ*_*n*0_, where *δ* is the Kronecker delta function (i.e. all cells start in the first microstate).

Assuming that microstates 0, 1, 2,…, *n*_*A*_ identify with the ESC state, microstates *n*_*A*_ + 1, *n*_*A*_ + 2,…, *n*_*B*_ identify with the EPI state, and microstates *n*_*B*_ + 1, *n*_*B*_ + 2,…, *N* identify with the NPC state, the observed probabilities are then

(Equation 17) $$p_{A}\left(t\right)=\sum_{n=0}^{n_{A}}p_{n}\left(t\right)\text{,}\;\;p_{B}\left(t\right)=\sum_{n=n_{A}+1}^{n_{B}}p_{n}\left(t\right)\text{,}\;\;p_{C}\left(t\right)=\sum_{n=n_{B}+1}^{N}p_{n}\left(t\right)\text{.}$$

#### Conveyor Belt Model

The hidden Markov model that we present in the main text assumes an initially homogeneous population and allows cell-cell variability to develop due to the inherently stochastic nature of the differentiation process. Although 2i conditions are known to produce a relatively pure population of robustly pluripotent cells, and we observe a general increase in cell-cell variability during differentiation (Figures 2G and 2H), it is possible that at least some of the variation seen during the differentiation process is due to deterministic propagation of initial cell-cell variability in ‘conveyor belt’-like process.

To model this we assume that differentiation progresses along a continuous one dimensional reaction coordinate *x*∈[−*L*,*L*] with initial population variability given by the probability density function *f*_0_(*x*). As differentiation progresses this initial variability propagates forward along the reaction coordinate *x* at constant speed *c*. The distribution of cell states at time *t* during the differentiation process is therefore given by *f*_*t*_ = *f*_0_(*x* + *ct*).

To account for the observed dynamics, we allow all cells at positions *x*∈*A* = [−*L*,*a*] to emit the ESC state, all cells at positions *x*∈*B* = [*a*,*b*] to emit the EPI state, and all cells at positions *x*∈*C* = [*b*,*L*] to emit the NPC state, where −*L* ≤*a* <*b* ≤ *L* are constants. The observed ESC, EPI and NPC probabilities are then,

(Equation 18) $$p_{A}\left(t\right)=F_{t}\left(a\right)\text{,}\;\;p_{B}\left(t\right)=F_{t}\left(a\right)-F_{t}\left(b\right)\text{,}\;\;p_{C}\left(t\right)=1-F_{t}\left(b\right)\text{,}$$

where *F*_*t*_(*x*) is the cumulative distribution function for *f*_*t*_(*x*).

To complete this model, we require a form for *f*_0_(*x*). There are two natural choices:

1. The initial variability is uniform on A. This is the equilibrium solution for diffusion of a Brownian particle on a bounded domain. Informally, this model assumes that when held in the naïve pluripotent state each cell takes an unbiased random walk on *A* and therefore no state within *A* is preferred. In this case, *f*_0_(*x*) = 1/(*a* + *L*) for *x*∈*A* and zero elsewhere, and the model has four free parameters: *a*, *b*, *c* and *L.*
2. The initial variability is Gaussian distributed on the domain [−∞,∞]. This is the equilibrium solution for an Ornstein-Uhlenbeck, or mean-reverting, process (i.e. diffusion of a Brownian particle on an infinite domain constrained by a quadratic potential). While the first variation assumes that there are no preferred states within the ESC state, the second model assumes that when held in the naïve pluripotent state individual cells are free to move randomly along the reaction coordinate, yet are continually drawn back to a ‘preferred’ configuration associated with the naïve ESC identity. In this case, $f_{0}\left(x\right)={\left(2\pi \sigma^{2}\right)}^{-\frac{1}{2}}\text{exp}\left(-{\left[x-\mu \right]}^{2}/2\sigma^{2}\right)$ and the model has five free parameters: *μ*, *σ*, *a*, *b* and *c.*

The first variation of this model predicts linear loss from the ESC and EPI states and so does not fit the data well (see Figure 4D). However, the second variation allows for the sigmoidal kinetics we observe and so provides a good fit to the data, albeit at the expense of a larger number of free parameters (Figure 4E).

#### Macroscopic Dynamics for Hidden Markov Model

To describe the macroscopic dynamics of our hidden Markov model we introduce the probability densities *ρ*_*A*_(*t*,*τ*), *ρ*_*B*_(*t*,*τ*), and *ρ*_*C*_(*t*,*τ*), where *τ* is a cell-intrinsic variable that records the length of time that an individual cell has spent in each macrostate. The observed proportion of cells in each state at experimental time *t* may then be obtained by integrating over these internal times. Thus,

(Equation 19) $$p_{A}\left(t\right)=\int_{0}^{t}\rho_{A}\left(t\text{,}\tau \right)d\tau \text{,}\;\;p_{B}\left(t\right)=\int_{0}^{t}\rho_{B}\left(t\text{,}\tau \right)d\tau \text{,}\;\;p_{C}\left(t\right)=\int_{0}^{t}\rho_{C}\left(t\text{,}\tau \right)d\tau \text{.}$$

The dynamics of *ρ*_*A*_, *ρ*_*B*_ and *ρ*_*C*_ are given by the following set of partial differential equations,

(Equation 20) $$\frac{\partial \rho_{A}}{\partial t}+\frac{\partial \rho_{A}}{\partial \tau }=-q^{-1}\mu_{A}\left(\tau \right)\rho_{A}\;\text{with}\;\rho_{A}\left(0\text{,}\tau \right)=\delta \left(\tau \right)\text{,}$$

(Equation 21) $$\frac{\partial \rho_{B}}{\partial t}+\frac{\partial \rho_{B}}{\partial \tau }=-q^{-1}\mu_{B}\left(\tau \right)\rho_{B}\;\text{with}\;\rho_{B}\left(t,0\right)=q^{-1}\int_{0}^{t}\mu_{A}\left(\tau \right)\rho_{A}d\tau \text{,}$$

(Equation 22) $$\frac{\partial \rho_{C}}{\partial t}+\frac{\partial \rho_{C}}{\partial \tau }=0\;\text{with}\;\rho_{C}\left(t,0\right)=q^{-1}\int_{0}^{t}\mu_{B}\left(\tau \right)\rho_{B}d\tau \text{,}$$

where *μ*_*A*_(*τ*) and *μ*_*B*_(*τ*) are the cumulative distribution functions for the wait times in the ESC and EPI macrostates respectively. In this case, since microscopic dynamics are given by a homogeneous Poisson process, the wait times in the ESC and EPI states are Erlang distributed and

(Equation 23) $$\mu_{A}\left(\tau \right)=1-\frac{1}{\text{Γ}\left(n_{A}\right)}\gamma \left(n_{A},q\tau \right)\text{,}$$

(Equation 24) $$\mu_{B}\left(\tau \right)=1-\frac{1}{\text{Γ}\left(n_{B}-n_{A}\right)}\gamma \left(n_{B}-n_{A},q\tau \right)\text{,}$$

where Γ is the Gamma function and *γ* is the incomplete Gamma function. The terms on the left hand sides of (Equation 20), (Equation 21), (Equation 22) account for cellular aging in each of the macrostates, while the right hand sides and boundary conditions account for transitions between macrostates. In the case that *n*_*A*_ = 0, and *n*_*B*_ = 1, the microstates and macrostates are coincident and the model reduces to (Equation 1), (Equation 2), (Equation 3).

### Data and Software Availability

#### Fluidigm Dynamic Arrays

Single cell gene expression data reported in this paper is available on Mendeley Data (http://dx.doi.org/10.17632/g2md5gbhz7.1).

#### Microarrays

Microarray data reported in this paper is available from ArrayExpress under accession number E-MTAB-5861.

## Author Contributions

Conceptualization, P.S.S., F.-J.M., and B.D.M.; Methodology, P.S.S., A.B., T.E.C., M.P.H.S., B.D.M., C.P.P., and S.D.H.; Formal Analysis, P.S.S., M.L., A.S., A.B., T.E.C., M.P.H.S., and B.D.M.; Investigation, P.S.S., R.C.G.S., and F.A.; Resources, B.D.M.; Data Curation, P.S.S.; Writing – Original Draft, P.S.S., A.B., B.D.M.; Writing – Review and Editing, all authors; Visualization, P.S.S. and B.D.M.; Supervision, B.D.M.; Project Administration, P.S.S. and B.D.M.; Funding Acquisition, B.D.M.

## Supplemental Information

Document S1. Figures S1–S3

Table S1. PhysioSpace Annotation, Related to Figures 1C and S1, Table S2, and STAR Methods List of global gene expression microarray data sourced from public repositories to construct the similarity score.

Table S2. PhysioSpace Results, Related to Figures 1C and S1 and Table S1 Similarity scores in column vector form for each time point.

Table S3. GO-Enrichment Results, Related to Figures S2F–S2H Results of Gene Ontology search for differentially expressed genes based on global gene expression microarrays.

Table S4. TaqMan Probes Used and Gene Annotation, Related to Figures 1 and 2 and STAR Methods List of oligonucleotides used in this study for single-cell gene expression arrays and corresponding gene annotation based on the literature.

Document S2. Article plus Supplemental Information

## References

[bib1] Abranches E., Guedes A.M.V., Moravec M., Maamar H., Svoboda P., Raj A., Henrique D. (2014). Stochastic NANOG fluctuations allow mouse embryonic stem cells to explore pluripotency. Development.

[bib2] Abranches E., Silva M., Pradier L., Schulz H., Hummel O., Henrique D., Bekman E. (2009). Neural differentiation of embryonic stem cells in vitro: a road map to neurogenesis in the embryo. PLoS One.

[bib3] Acampora D., Di Giovannantonio L.G., Simeone A. (2013). Otx2 is an intrinsic determinant of the embryonic stem cell state and is required for transition to a stable epiblast stem cell condition. Development.

[bib4] Bain G., Ray W.J., Yao M., Gottlieb D.I. (1996). Retinoic acid promotes neural and represses mesodermal gene expression in mouse embryonic stem cells in culture. Biochem. Biophys. Res. Commun..

[bib5] Bedzhov I., Zernicka-Goetz M. (2014). Self-organizing properties of mouse pluripotent cells initiate morphogenesis upon implantation. Cell.

[bib6] Betschinger J., Nichols J., Dietmann S., Corrin P.D., Paddison P.J., Smith A. (2013). Exit from pluripotency is gated by intracellular redistribution of the bHLH transcription factor Tfe3. Cell.

[bib7] Boroviak T., Loos R., Bertone P., Smith A., Nichols J. (2014). The ability of inner-cell-mass cells to self-renew as embryonic stem cells is acquired following epiblast specification. Nat. Cell Biol..

[bib8] Broccoli V., Boncinelli E., Wurst W. (1999). The caudal limit of Otx2 expression positions the isthmic organizer. Nature.

[bib9] Cell Systems (2017). What is your conceptual definition of “cell type” in the context of a mature organism?. Cell Syst..

[bib10] Chambers I., Silva J., Colby D., Nichols J., Nijmeijer B., Robertson M., Vrana J., Jones K., Grotewold L., Smith A. (2007). Nanog safeguards pluripotency and mediates germline development. Nature.

[bib11] Chan T.E., Stumpf M., Babtie A.C. (2017). Network inference and hypotheses-generation from single-cell transcriptomic data using multivariate information measures. Cell Syst..

[bib12] Chang H.H., Hemberg M., Barahona M., Ingber D.E., Huang S. (2008). Transcriptome-wide noise controls lineage choice in mammalian progenitor cells. Nature.

[bib13] Delvenne J.C., Yaliraki S.N., Barahona M. (2010). Stability of graph communities across time scales. Proc. Natl. Acad. Sci. USA.

[bib14] Deng Q., Ramsköld D., Reinius B., Sandberg R. (2014). Single-cell RNA-seq reveals dynamic, random monoallelic gene expression in mammalian cells. Science.

[bib15] Doetschman T., Gregg R.G., Maeda N., Hooper M.L., Melton D.W., Thompson S., Smithies O. (1987). Targetted correction of a mutant HPRT gene in mouse embryonic stem cells. Nature.

[bib16] Du P., Kibbe W.A., Lin S.M. (2008). lumi: a pipeline for processing Illumina microarray. Bioinformatics.

[bib17] Dunn S.-J., Martello G., Yordanov B., Emmott S., Smith A.G. (2014). Defining an essential transcription factor program for naïve pluripotency. Science.

[bib18] Furusawa C., Kaneko K. (2012). A dynamical-systems view of stem cell biology. Science.

[bib19] Garcia-Ojalvo J., Martinez-Arias A. (2012). Towards a statistical mechanics of cell fate decisions. Curr. Opin. Genet. Dev..

[bib20] Gardiner C.W. (1985). Handbook of Stochastic Methods.

[bib21] Gower J. (1974). Algorithm AS 78: the mediancentre. J. R. Stat. Soc. Series C Appl. Stat.

[bib22] Habib S.J., Chen B.-C., Tsai F.-C., Anastassiadis K., Meyer T., Betzig E., Nusse R. (2013). A localized Wnt signal orients asymmetric stem cell division in vitro. Science.

[bib23] Habibi E., Brinkman A.B., Arand J., Kroeze L.I., Kerstens H.H.D., Matarese F., Lepikhov K., Gut M., Brun-Heath I., Hubner N.C. (2013). Whole-genome bisulfite sequencing of two distinct interconvertible DNA methylomes of mouse embryonic stem cells. Cell Stem Cell.

[bib24] Hastie T., Tibshirani R., Friedman J. (2001). The Elements of Statistical Learning: Data Mining, Inference, and Prediction, Springer Series in Statistics.

[bib25] Hayashi K., de Sousa Lopes S.M.C., Lopes S.M.C., Tang F., Lao K., Surani M.A. (2008). Dynamic equilibrium and heterogeneity of mouse pluripotent stem cells with distinct functional and epigenetic states. Cell Stem Cell.

[bib26] Hormoz S., Singer Z.S., Linton J.M., Antebi Y.E., Shraiman B.I., Elowitz M.B. (2016). Inferring cell-state transition dynamics from lineage trees and endpoint single-cell measurements. Cell Syst..

[bib27] Kalkan T., Smith A. (2014). Mapping the route from naive pluripotency to lineage specification. Philos. Trans. R. Soc. Lond. B, Biol. Sci..

[bib28] Kuehn M.R., Bradley A., Robertson E.J., Evans M.J. (1987). A potential animal model for Lesch-Nyhan syndrome through introduction of HPRT mutations into mice. Nature.

[bib29] Lawson C.L., Hanson R.J. (1995). Solving Least Squares Problems.

[bib30] Lee H.J., Hore T.A., Reik W. (2014). Reprogramming the methylome: erasing memory and creating diversity. Cell Stem Cell.

[bib31] Lenz M., Schuldt B.M., Müller F.-J., Schuppert A. (2013). PhysioSpace: relating gene expression experiments from heterogeneous sources using shared physiological processes. PLoS One.

[bib32] MacArthur B.D., Lemischka I.R. (2013). Statistical mechanics of pluripotency. Cell.

[bib33] MacArthur B.D., Ma’ayan A., Lemischka I.R. (2009). Systems biology of stem cell fate and cellular reprogramming. Nat. Rev. Mol. Cell Biol..

[bib34] MacArthur B.D., Sevilla A., Lenz M., Müller F.-J., Schuldt B.M., Schuppert A.A., Ridden S.J., Stumpf P.S., Fidalgo M., Ma’ayan A. (2012). Nanog-dependent feedback loops regulate murine embryonic stem cell heterogeneity. Nat. Cell Biol..

[bib35] Marks H., Kalkan T., Menafra R., Denissov S., Jones K., Hofemeister H., Nichols J., Kranz A., Stewart A.F., Smith A., Stunnenberg H.G. (2012). The transcriptional and epigenomic foundations of ground state pluripotency. Cell.

[bib36] McMahon S.S., Sim A., Johnson R., Liepe J., Stumpf M.P.H. (2014). Information theory and signal transduction systems: from molecular information processing to network inference. Semin. Cell Dev. Biol..

[bib37] Meissner A., Mikkelsen T.S., Gu H., Wernig M., Hanna J., Sivachenko A., Zhang X., Bernstein B.E., Nusbaum C., Jaffe D.B. (2008). Genome-scale DNA methylation maps of pluripotent and differentiated cells. Nature.

[bib38] Mikkelsen T.S., Ku M., Jaffe D.B., Issac B., Lieberman E., Giannoukos G., Alvarez P., Brockman W., Kim T.-K., Koche R.P. (2007). Genome-wide maps of chromatin state in pluripotent and lineage-committed cells. Nature.

[bib39] Millett S., Campbell K., Epstein D.J., Losos K., Harris E., Joyner A.L. (1999). A role for Gbx2 in repression of Otx2 and positioning the mid/hindbrain organizer. Nature.

[bib40] Miyanari Y., Torres-Padilla M.-E. (2012). Control of ground-state pluripotency by allelic regulation of Nanog. Nature.

[bib41] Mojtahedi M., Skupin A., Zhou J., Castaño I.G., Leong-Quong R.Y.Y., Chang H., Trachana K., Giuliani A., Huang S. (2016). Cell fate decision as high-dimensional critical state transition. PLoS Biol..

[bib42] Moris N., Pina C., Martinez-Arias A. (2016). Transition states and cell fate decisions in epigenetic landscapes. Nat. Rev. Genet..

[bib43] Müller F.-J., Laurent L.C., Kostka D., Ulitsky I., Williams R., Lu C., Park I.-H., Rao M.S., Shamir R., Schwartz P.H. (2008). Regulatory networks define phenotypic classes of human stem cell lines. Nature.

[bib44] Nagy A., Rossant J., Nagy R., Abramow-Newerly W., Roder J.C. (1993). Derivation of completely cell culture-derived mice from early-passage embryonic stem cells. Proc. Natl. Acad. Sci. USA.

[bib45] Newman M. (2010). Networks: An Introduction.

[bib46] Nichols J., Smith A. (2009). Naive and primed pluripotent states. Cell Stem Cell.

[bib47] Pathria R.K. (1996). Statistical Mechanics.

[bib48] Richard A., Boullu L., Herbach U., Bonnafoux A., Morin V., Vallin E., Guillemin A., Papili Gao N., Gunawan R., Cosette J. (2016). Single-cell-based analysis highlights a surge in cell-to-cell molecular variability preceding irreversible commitment in a differentiation process. PLoS Biol..

[bib49] Ridden S.J., Chang H.H., Zygalakis K.C., MacArthur B.D. (2015). Entropy, ergodicity, and stem cell multipotency. Phys. Rev. Lett..

[bib50] Salomonis N., Schlieve C.R., Pereira L., Wahlquist C., Colas A., Zambon A.C., Vranizan K., Spindler M.J., Pico A.R., Cline M.S. (2010). Alternative splicing regulates mouse embryonic stem cell pluripotency and differentiation. Proc. Natl. Acad. Sci. USA.

[bib51] Scargle J.D., Norris J.P., Jackson B., Chiang J. (2013). Studies in astronomical time series analysis. VI. Bayesian block representations. Astrophys. J..

[bib52] Scheffer M., Bascompte J., Brock W.A., Brovkin V., Carpenter S.R., Dakos V., Held H., van Nes E.H., Rietkerk M., Sugihara G. (2009). Early-warning signals for critical transitions. Nature.

[bib53] Semrau S., Goldmann J., Soumillon M., Mikkelsen T.S., Jaenisch R., van Oudenaarden A. (2016). Dynamics of lineage commitment revealed by single-cell transcriptomics of differentiating embryonic stem cells. bioRxiv.

[bib54] Shapiro E., Biezuner T., Linnarsson S. (2013). Single-cell sequencing-based technologies will revolutionize whole-organism science. Nat. Rev. Genet..

[bib55] Simeone A., Acampora D., Gulisano M., Stornaiuolo A., Boncinelli E. (1992). Nested expression domains of four homeobox genes in developing rostral brain. Nature.

[bib56] Singer Z.S., Yong J., Tischler J., Hackett J.A., Altinok A., Surani M.A., Cai L., Elowitz M.B. (2014). Dynamic heterogeneity and DNA methylation in embryonic stem cells. Mol. Cell.

[bib57] Stainier D.Y.R., Gilbert W. (1990). Pioneer neurons in the mouse trigeminal sensory system. Proc. Natl. Acad. Sci. USA.

[bib58] Stegle O., Teichmann S.A., Marioni J.C. (2015). Computational and analytical challenges in single-cell transcriptomics. Nat. Rev. Genet..

[bib59] Tam P.P.L., Loebel D.A.F. (2007). Gene function in mouse embryogenesis: get set for gastrulation. Nat. Rev. Genet..

[bib60] Tibshirani R., Walther G., Hastie T. (2001). Estimating the number of clusters in a data set via the gap statistic. J. R. Stat. Soc. Series B Stat. Methodol..

[bib61] Timme N., Alford W., Flecker B., Beggs J.M. (2014). Synergy, redundancy, and multivariate information measures: an experimentalist’s perspective. J. Comput. Neurosci..

[bib62] Toyooka Y., Shimosato D., Murakami K., Takahashi K., Niwa H. (2008). Identification and characterization of subpopulations in undifferentiated ES cell culture. Development.

[bib63] Trott J., Hayashi K., Surani A., Babu M.M., Martinez-Arias A. (2012). Dissecting ensemble networks in ES cell populations reveals micro-heterogeneity underlying pluripotency. Mol. Biosyst..

[bib64] Tuna S., Niranjan M. (2010). Reducing the algorithmic variability in transcriptome-based inference. Bioinformatics.

[bib65] Turner D.A., Trott J., Hayward P., Rué P., Martinez-Arias A. (2014). An interplay between extracellular signalling and the dynamics of the exit from pluripotency drives cell fate decisions in mouse ES cells. Biol. Open.

[bib66] van den Brink S.C., Baillie-Johnson P., Balayo T., Hadjantonakis A.-K., Nowotschin S., Turner D.A., Martinez-Arias A. (2014). Symmetry breaking, germ layer specification and axial organisation in aggregates of mouse embryonic stem cells. Development.

[bib67] Williams P.L., Beer R.D. (2010). Nonnegative decomposition of multivariate information. arxiv.

[bib68] Ying Q.-L., Stavridis M., Griffiths D., Li M., Smith A. (2003). Conversion of embryonic stem cells into neuroectodermal precursors in adherent monoculture. Nat. Biotechnol..

[bib69] Ying Q.-L., Wray J., Nichols J., Batlle-Morera L., Doble B., Woodgett J., Cohen P., Smith A. (2008). The ground state of embryonic stem cell self-renewal. Nature.

[bib70] Ziller M.J., Edri R., Yaffe Y., Donaghey J., Pop R., Mallard W., Issner R., Gifford C.A., Goren A., Xing J. (2015). Dissecting neural differentiation regulatory networks through epigenetic footprinting. Nature.

