# Supplementary material for: Stem Cell Differentiation as a Non-Markov Stochastic Process
Source: Cell Syst. 2017 Sep 27;5(3):268–282.e7. doi: 10.1016/j.cels.2017.08.009 (PMC5624514; doi:10.1016/j.cels.2017.08.009)
Supplement: Document S1. Figures S1–S3 [file mmc1.pdf]

**Cell Systems, Volume *5***

## Supplemental Information

# Stem Cell Differentiation as a Non-Markov Stochastic Process

**Patrick S. Stumpf, Rosanna C.G. Smith, Michael Lenz, Andreas Schuppert, Franz-Josef Müller, Ann Babtie, Thalia E. Chan, Michael P.H. Stumpf, Colin P. Please, Sam D. Howison, Fumio Arai, and Ben D. MacArthur**

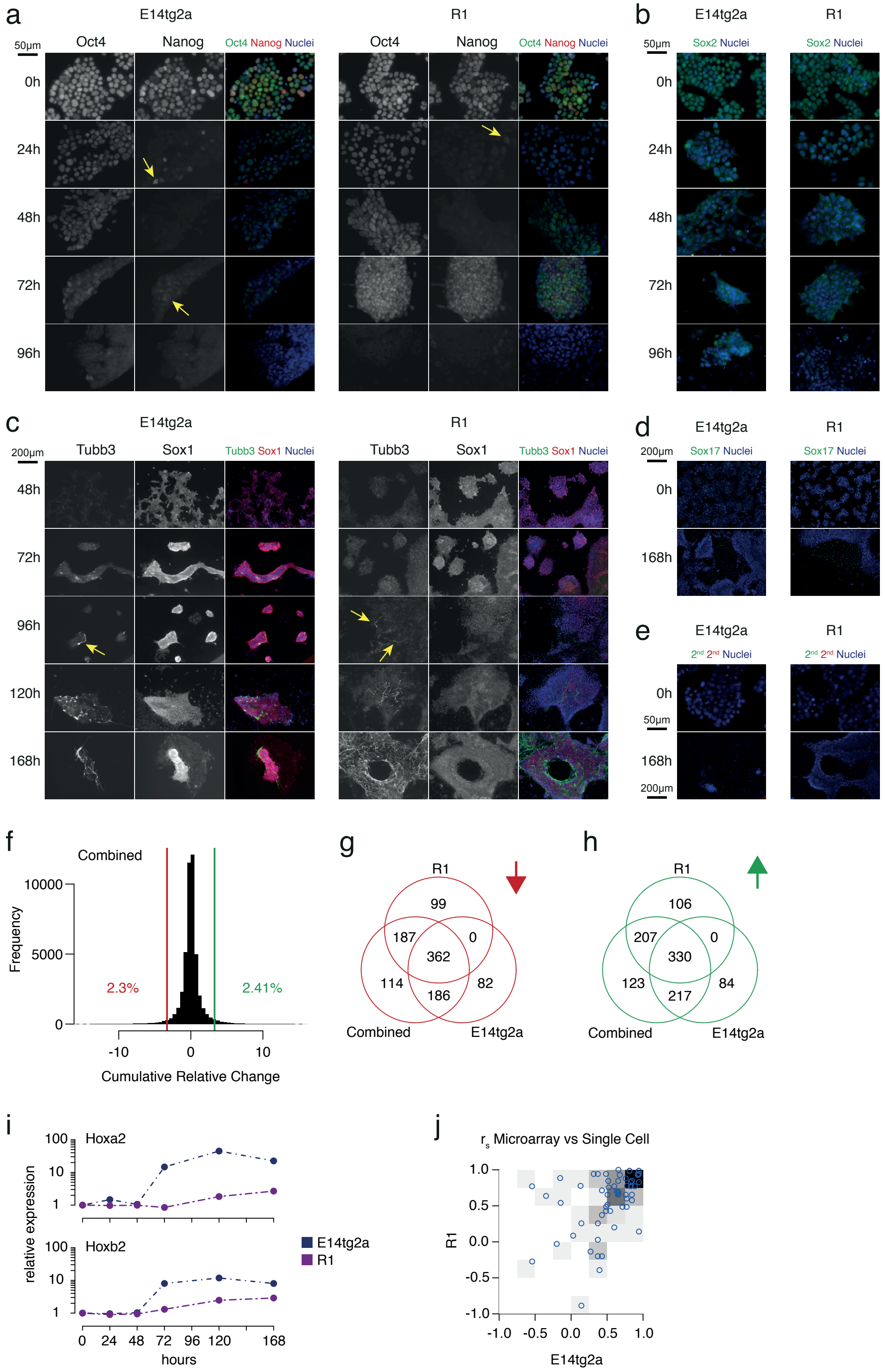


Figure S1: **Expression changes during time-course - Related to Fig. 1**. (a-e) Protein expression dynamics of (a) pluripotency associated transcription factors Oct4 and Nanog and (b) Sox2, (c) neuroectoderm markers Tubb3 and Sox1, (d) endodermal marker Sox17. Arrows show residual cells expressing Nanog and pioneer neurons respectively (e) Negative control. (f) Histogram of microarray-based expression changes across both E14 and R1 cells. (g, h) Venn diagram of (g) down- and (h) up-regulated genes. (i) Relative expression changes of Hoxa2 and Hoxb2. (j) Rank-correlation coefficient of average single-cell versus ensemble cell expression for R1 and E14 cells over time.

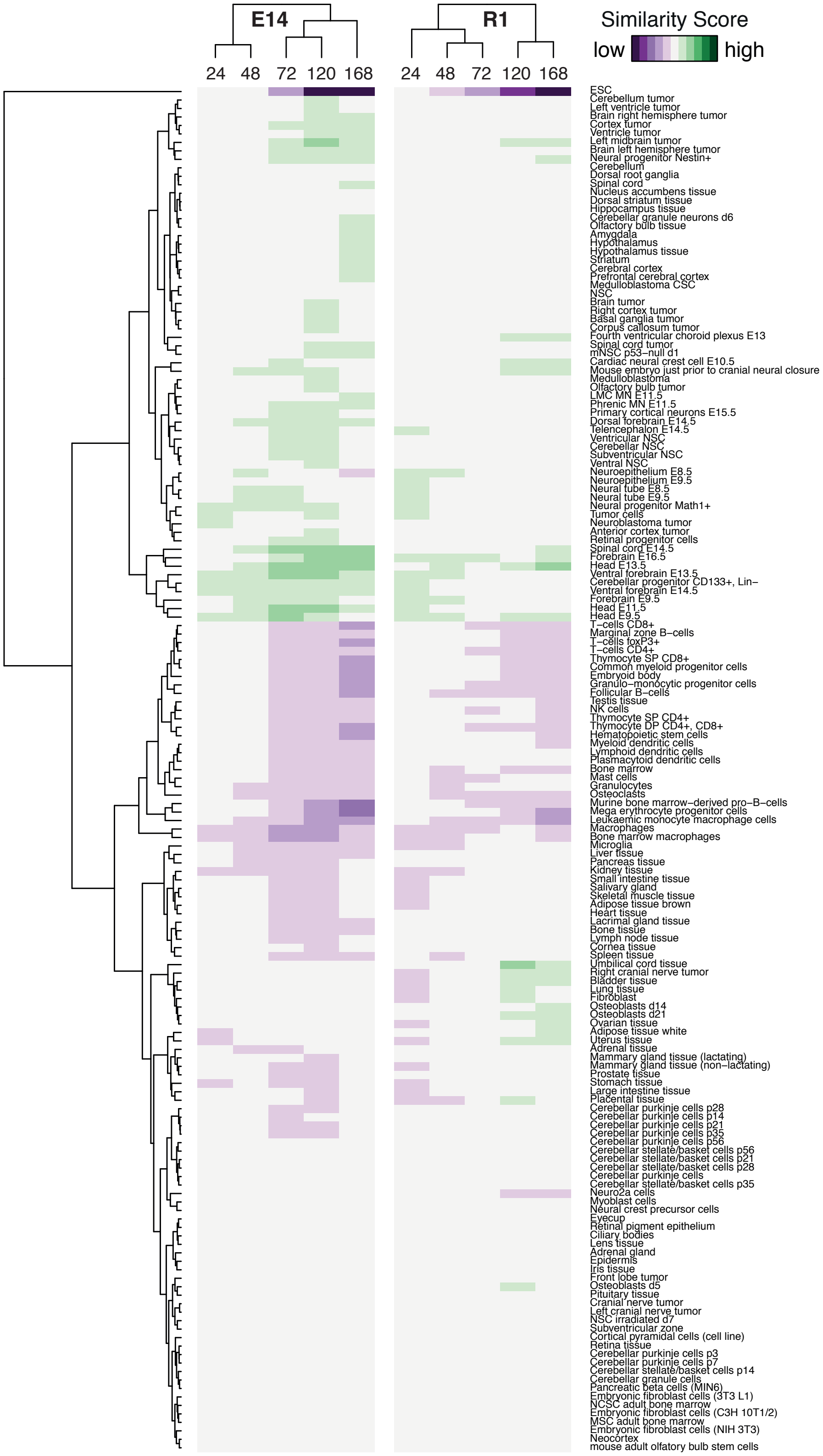


Figure S2: **PhysioSpace similarity scores - Related to Fig. 1c**. Similarity of time-course data for all of the 161 lineages we considered (see Supplementary Table S1 for a full list datasets used in this comparison and Supplementary Table S2 for similarity data).

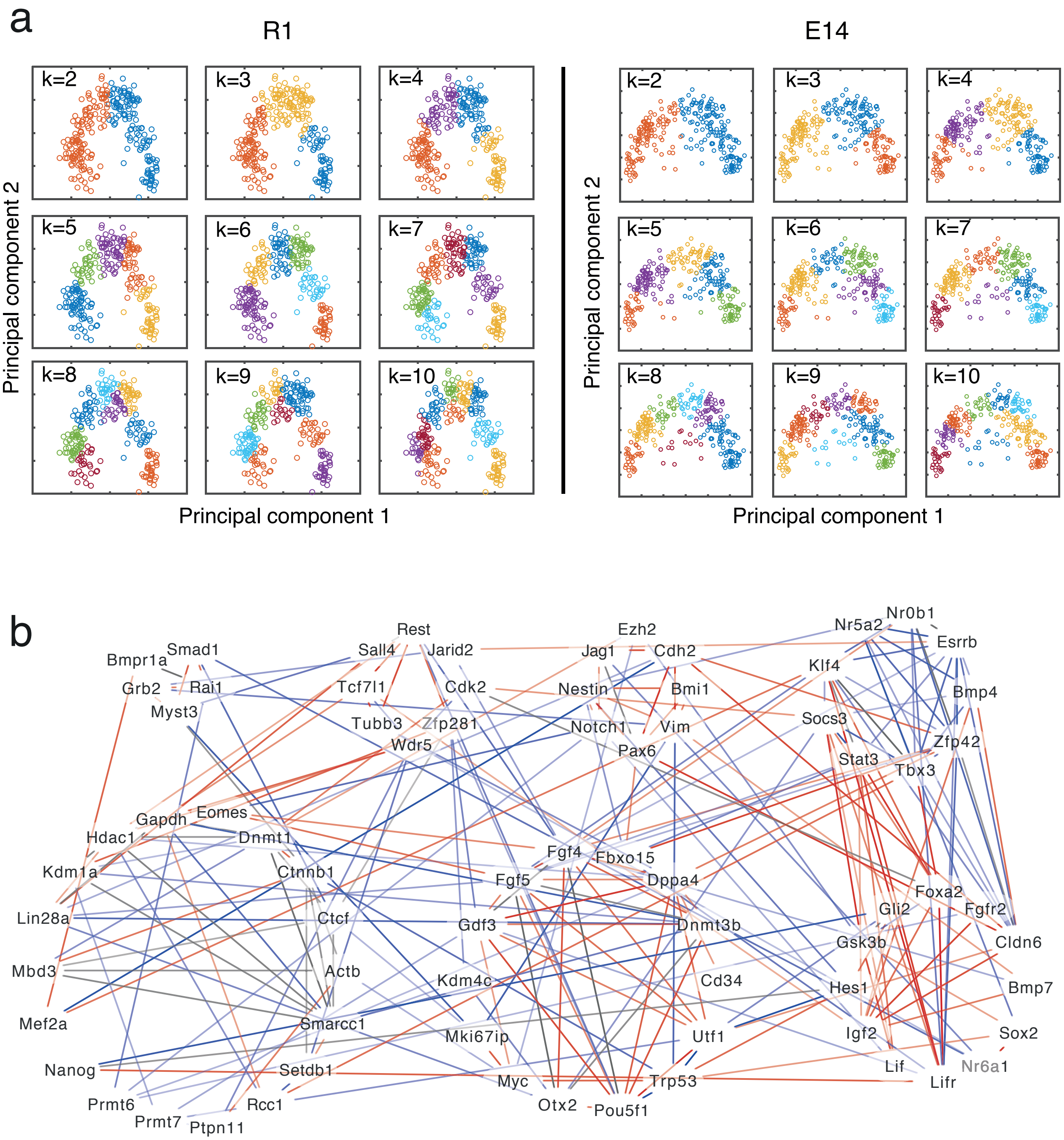


Figure S3: **Single-cell classification and network inference - Related to Fig. 2**. (a) PCA plot of k-means clustering with k from 2-10. (b) (Co-)regulatory network inferred from single-cell data. Genes naturally grouped into seven modules based on an unbiased community detection algorithm (See main text for details). Significant interactions in cells classified as ESC or EPI are in blue, while significant interactions in cells classified as EPI or NPC are in red.

**Table S1: PhysioSpace annotation - Related to Fig. 1c, Fig. S1, Table S2 and STAR Methods.** List of global gene expression microarray data sourced from public repositories to construct the similarity score.

**Table S2: PhysioSpace results - Related to Fig. 1c, Fig. S1 and Table S1.** Similarity scores in column vector form for each time point.

**Table S3: GO-enrichment results - Related to Fig. S2f-h.** Results of Gene Ontology search for differentially expressed genes based on global gene expression microarrays.

**Table S4: TaqMan probes used and gene annotation - Related to Fig. 1, Fig.2 and STAR Methods.** List of oligonucleotides used in this study for single-cell gene expression arrays and corresponding gene annotation based on the literature.
